# The Next-Generation Oral Selective Estrogen Receptor Degrader Camizestrant (AZD9833) Suppresses ER^+^ Breast Cancer Growth and Overcomes Endocrine and CDK4/6 Inhibitor Resistance

**DOI:** 10.1158/0008-5472.CAN-23-0694

**Published:** 2023-09-19

**Authors:** Mandy Lawson, Natalie Cureton, Susana Ros, Azadeh Cheraghchi-Bashi, Jelena Urosevic, Sophie D'Arcy, Oona Delpuech, Michelle DuPont, David I. Fisher, Eric T. Gangl, Hilary Lewis, Dawn Trueman, Neha Wali, Stuart C. Williamson, Jennifer Moss, Elodie Montaudon, Heloise Derrien, Elisabetta Marangoni, Ricardo J. Miragaia, Sladjana Gagrica, Pablo Morentin-Gutierrez, Thomas A. Moss, Gareth Maglennon, Daniel Sutton, Radoslaw Polanski, Alan Rosen, Jonathan Cairns, Pei Zhang, Mònica Sánchez-Guixé, Violeta Serra, Susan E. Critchlow, James S. Scott, Justin P.O. Lindemann, Simon T. Barry, Teresa Klinowska, Christopher J. Morrow, Larissa S. Carnevalli

**Affiliations:** 1 The Discovery Centre, Biomedical Campus, AstraZeneca, Cambridge, United Kingdom.; 2 Research and Early Development, Oncology R&D, AstraZeneca, Waltham, Massachusetts.; 3 Discovery Sciences, Biopharmaceuticals R&D, AstraZeneca, Cambridge, United Kingdom.; 4 Institut Curie, Paris, France.; 5 Oncology Data Science, Oncology R&D, AstraZeneca, Cambridge, United Kingdom.; 6 Clinical Pharmacology and Safety Sciences, Biopharmaceuticals R&D, AstraZeneca, Cambridge, United Kingdom.; 7 Experimental Therapeutics Group, Vall d'Hebron Institute of Oncology (VHIO), Barcelona, Spain.; 8 Late Development, Oncology R&D, AstraZeneca, Cambridge, United Kingdom.

## Abstract

Oral selective estrogen receptor degraders (SERD) could become the backbone of endocrine therapy (ET) for estrogen receptor–positive (ER^+^) breast cancer, as they achieve greater inhibition of ER-driven cancers than current ETs and overcome key resistance mechanisms. In this study, we evaluated the preclinical pharmacology and efficacy of the next-generation oral SERD camizestrant (AZD9833) and assessed ER–co-targeting strategies by combining camizestrant with CDK4/6 inhibitors (CDK4/6i) and PI3K/AKT/mTOR-targeted therapy in models of progression on CDK4/6i and/or ET. Camizestrant demonstrated robust and selective ER degradation, modulated ER-regulated gene expression, and induced complete ER antagonism and significant antiproliferation activity in *ESR1* wild-type (*ESR1*wt) and mutant (*ESR1*m) breast cancer cell lines and patient-derived xenograft (PDX) models. Camizestrant also delivered strong antitumor activity in fulvestrant-resistant *ESR1*wt and *ESR1*m PDX models. Evaluation of camizestrant in combination with CDK4/6i (palbociclib or abemaciclib) in CDK4/6-naive and -resistant models, as well as in combination with PI3Kαi (alpelisib), mTORi (everolimus), or AKTi (capivasertib), indicated that camizestrant was active with CDK4/6i or PI3K/AKT/mTORi and that antitumor activity was further increased by the triple combination. The response was observed independently of PI3K pathway mutation status. Overall, camizestrant shows strong and broad antitumor activity in ER^+^ breast cancer as a monotherapy and when combined with CDK4/6i and PI3K/AKT/mTORi.

**Significance:**

Camizestrant, a next-generation oral SERD, shows promise in preclinical models of ER^+^ breast cancer alone and in combination with CDK4/6 and PI3K/AKT/mTOR inhibitors to address endocrine resistance, a current barrier to treatment.

## Introduction

Estrogen contributes significantly to many breast cancers by regulating the growth and differentiation of breast epithelial cells. This is mediated through its interaction with estrogen receptor alpha (ERα): A ligand-activated transcription factor encoded by the *ER-1* (*ESR1*) gene (1). Around 70% to 80% of patients with breast cancer have hormone receptor–positive (HR^+^) disease—meaning that their tumors express ER and/or progesterone receptor (PgR)—and most of these have ER-driven tumors at diagnosis (1). Thus, endocrine therapies (ET) affecting ER-driven proliferation are routinely prescribed for such patients (1).

ETs for breast cancer include selective ER modulators (SERMs; e.g., tamoxifen); gonadotropin-releasing hormone agonists (e.g., goserelin); aromatase inhibitors (AI; e.g., anastrozole, letrozole, exemestane); and the selective ER antagonist and degrader (SERD, selective estrogen receptor degrader) fulvestrant: The only SERD approved for first- and second-line metastatic ER^+^ (i.e., ER expressing) breast cancer (2–4).

Although these agents effectively disrupt ER signaling, many are limited by intrinsic or acquired drug resistance. For example, SERMs can block the activation function (AF) 2 domain but not the AF1 domain of ER (5), agonizing certain tissues (e.g., uterus), which can, in other contexts, stimulate tumor growth (6). Similarly, AI treatment can lead to clonal outgrowth of cells harboring resistance mutations in *ESR1* (*ESR1*m), which constitutively activate ER (1).

Despite this resistance, tumors often continue depending on ER activity for growth (7). Indeed, addressing ET resistance is the most significant unmet need in patients with HR^+^ breast cancer (1).

Adding cyclin-dependent kinase 4/6 inhibitors (CDK4/6i) to ET significantly improves outcomes for patients with metastatic HR^+^ breast cancer, prolonging progression-free survival (PFS; refs. 8, 9). However, CDK4/6i resistance can also occur, with several cellular mechanisms described previously. These include upregulation of the PI3K/AKT/mTOR signaling pathway (10). Moreover, approximately 40% of HR^+^ tumors also carry a *PIK3CA* (PI3K subunit p110α) mutation, constitutively activating the PI3K/AKT/mTOR pathway (11, 12). Accordingly, simultaneously blocking the ER and PI3K pathways heightens antitumor activity in these subpopulations (13). Constitutive activation of the PI3K/AKT/mTOR pathway sometimes occurs via activating mutations in *AKT1* or loss-of-function alterations in negative regulator PTEN (12). This led to the strategy of combining ET not only with CDK4/6 (e.g., palbociclib) or PI3Kα (e.g., alpelisib) inhibitors, but also with mTOR (e.g., everolimus) or AKT (e.g., capivasertib) inhibitors (14). Furthermore, triple combination of PI3K or AKT inhibitors with CDK4/6i and fulvestrant significantly inhibits the growth of patient-derived xenografts (PDX) resistant to double therapy alone (14, 15).

SERDs compete with estrogen for ER binding, antagonizing, and degrading ER whether ligand-driven or constitutively active because of *ESR1* mutations suppressing ER-dependent signaling (16, 17). Fulvestrant is the first-in-class SERD and, because it lacks agonism in all ER^+^ tissues, the first pure ER antagonist (18). It is effective in patients with ER^+^ breast cancer, whether naïve or resistant to tamoxifen and AIs (18, 19). However, fulvestrant has low oral bioavailability, mandating invasive and uncomfortable intramuscular administration, which limits the deliverable dose (20). Clinical studies with fulvestrant highlight the potential for greater ER degradation if higher exposure could be achieved (19, 21). However, the plasmaMATCH study showed that even at an effective intramuscular dose of 1,000 mg monthly, fulvestrant drove no clinical benefit in heavily pretreated patients with *ESR1*m advanced breast cancer (22).

To address fulvestrant's limitations, several orally bioavailable SERDs have been developed. AZD9496, AstraZeneca's first-generation oral SERD, exhibited ER degradation and antagonism comparable with fulvestrant, and with greater antitumor activity, in MCF7 xenografts (23, 24). However, its antitumor activity did not translate to other models.

More recently, other oral SERDs have entered clinical trials, including giredestrant (Roche; ref. 25), imlunestrant (Lilly; ref. 26), and amcenestrant (Sanofi; ref. 27), although amcenestrant's development was discontinued for poor efficacy (28). The SERM/SERD elacestrant (Radius/Menarini) showed modest benefit over standard-of-care ETs in patients with late-line metastatic breast cancer, with greatest median PFS uplift in patients with *ESR1*m disease (29). In addition, next-generation SERMs (e.g., lasofoxifene, bazedoxifene) are being developed as monotherapies and combined with CDK4/6i for advanced metastatic disease (30).

Here, we describe the preclinical characterization of the next-generation SERD (ngSERD) camizestrant (AZD9833). The data suggest that camizestrant's activity profile is superior to fulvestrant *in vivo*, with broad efficacy when combined with CDK4/6i and/or PI3K/AKT/mTOR inhibitors in models of early disease and primary/acquired resistance to ETs and CDK4/6i.

## Materials and Methods

### Gene expression analysis in cell lines and PDX models

MCF7 and CAMA-1 cells were seeded into 6-well microplates in phenol red-free RPMI-1640 medium (Invitrogen) containing 2 mmol/L l-glutamine and 5% (volume/volume, v/v) charcoal-stripped FBS (Sigma). Cells were treated with 1 nmol/L estradiol and 100 nmol/L fulvestrant, AZD9496, or camizestrant for 24 hours, with three replicates per condition and non–estradiol-treated controls. Samples were harvested in 1 mL of QIAzol lysis buffer and snap-frozen. Snap-frozen tissue from PDX studies was homogenized and resuspended in QIAzol lysis buffer. RNA was extracted using RNeasy 96 QIAcube HT total RNA Cell with DNAse treatment, and samples randomized over 96-well plates. RNA was quantified using the Qubit RNA BR kit (Q10213 Invitrogen), per the manufacturer's instructions.

### Analysis of RNA integrity, quality control, and gene expression quantification of RNA sequencing

After sequencing, RNA integrity was analyzed on a TapeStation 4200 using the RNA 6000 Nano Kit (5067 1511, Agilent), according to the manufacturers’ instructions. The Illumina Truseq stranded mRNA library was generated by the Cancer Research UK Cambridge Institute Genomic core facility, and sequencing of cell lines was performed on the Illumina Hiseq4000 platform, providing single-end 50 base pair reads and around 20 million reads per sample. PDX samples were sequenced using Illumina NovaSeq600, providing paired-end 100 base pair reads and around 34 million reads per sample.

Quality control and gene expression quantification of RNA sequencing (RNA-seq) were performed using the RNA-seq pipeline implemented in bcbio-nextgen (https://bcbio-nextgen.readthedocs.org/en/latest/). Reads were aligned to the University of California (Santa Cruz, CA) *Homo sapiens* GRCh38 genome build, augmented with transcript information from Ensembl release 86 using HiSat2 (31). Alignments were evaluated for evenness of coverage, ribosomal RNA content, genomic context of alignments, and complexity, using a combination of FastQC, Qualimap, and custom computational tools (32). Transcripts per million measurements per isoform were generated by quasi-alignment using Salmon and were used to estimate the abundance of genes (33). The aggregated gene counts were used for differential gene expression analyses using DESeq2 (34).

### Proliferation assays

Camizestrant's ability to inhibit proliferation *in vitro* was measured in ER^+^ breast cancer cell lines using the Sytox Green live/dead cell count. MCF7 and CAMA-1 cells were seeded into 96-well microplates in phenol red-free RPMI-1640 medium containing 2 mmol/L l-glutamine and 5% (v/v) charcoal-stripped FBS at 4×10^3^ and 8×10^3^ cells/well, based on the doubling time of each cell line. Plates were incubated for 24 hours (37°C; 5% CO_2_) and a 10-point concentration range of selected compounds was dispensed onto the cells. Dimethyl sulfoxide (DMSO) was dispensed into control wells to give a 0.1% final concentration. One plate was untreated, as a day 0 control. Live cells were counted at days 0 and 7 (or day 6 for Y537S *ESR1*m MCF7 cells) using Sytox Green nucleic assay stain (180 nmol/L per well). Green (dead) cells were counted at days 0 and 7 using an Acumen Explorer (TTP Labtech) or Cellinsight imager (Thermo Fisher Scientific). Saponin 0.045% (weight/volume) was added to permeabilize the cells overnight, permitting total cell count. The number of live cells was calculated by subtracting dead cells from the total cell count, and curve-fitted using GraphPad Prism. Average cell counts from the day 0 plate were used to determine 0% cell growth. A dose–response curve was plotted using non-linear regression to determine the IC_50_ and bottom-of-curve values. Viability assay methods are described in Supplementary Methods.

### ER degradation

Camizestrant's ability to degrade ER was assessed using ER^+^ human breast cancer cell lines (MCF7, CAMA-1, BT474, ZR-75–1, T47D, and MDA-MB-361) and an ER^+^ human endometrial cell line (Ishikawa). Cells were seeded into 12-well tissue culture-treated plates (0.5×10^6^ cells/well) in phenol red-free RPMI medium containing 5% (v/v) charcoal-stripped FBS. Cells were incubated for 48 hours (37°C; 5% CO_2_) with 100 nmol/L camizestrant, AZD9496, or fulvestrant.

Cell lysates were loaded on NuPAGE 4% to 12% Bis-Tris Midi Protein Gels, transferred to nitrocellulose membranes and immunoblotted with anti-ER antibody (clone SP1, Thermo Fisher Scientific). To detect ER agonism, Ishikawa cells were incubated in media containing 5% (v/v) double charcoal-stripped FBS, achieved by stripping FBS using activated charcoal (C9157, Sigma). Blots were probed with anti-PgR antibody (clone 636, Dako) then horseradish peroxidase secondary antibody (anti-mouse, 7076 Cell Signaling Technology or anti-rabbit, 7070 Cell Signaling Technology). Protein levels were measured on the G-box using Pierce West Dura and West Femto chemiluminescent reagents and Syngene software.

### *In vivo* models, patient-derived cells, and PDX models

All animal experiments were conducted in accordance with UK Home Office legislation, the Animal Scientific Procedures Act 1986, and the AstraZeneca Global Bioethics policy. All experimental work done at AstraZeneca is outlined in project licenses P0EC1FFDF or PP3292652, which went through an Animal Welfare and Ethical Review Board, followed by approval by the Home Office. All experiments followed the principles of good statistical practice, as well as the PREPARE and ARRIVE guidelines. AstraZeneca is a signatory to the Concordat on Openness on Animal Research in the UK. All animal studies were conducted by contract research organizations in accordance with local authorities, guidelines of the Animal Welfare Act, and the AstraZeneca Global Bioethics policy. Animal studies were performed in accordance with protocols approved by the START “Institutional Animal Care and Use Committee” along with the AstraZeneca's “Platform for Animal Research Tracking and External Relationships” (PARTNER) group.

#### PDX models

For all PDX models, tumors were measured twice weekly; changes in tumor volume and growth inhibition were determined by bilateral Vernier caliper measurement (length x width). Length was the longest diameter across the tumor and width the corresponding perpendicular.

Details of formulating camizestrant, fulvestrant, elacestrant, palbociclib, abemaciclib, everolimus, capivasertib, and alpelisib appear in the Supplementary Methods, together with methods of establishing and harvesting patient-derived tumor tissue from mice.

For efficacy studies, animals were randomized into treatment groups and dosing started once tumor volume reached 150–300 mm^3^. Details of efficacy studies, and group sizes and treatments are described in corresponding figure legends. PDX models in Fig. 6 and Supplementary Fig. S1 were part of the multi-arm study (with arms irrelevant to this publication) with common control monotherapy arms (e.g., the vehicle-treated group). Hence, the same control data will be used in different publications.

Tumor growth inhibition from study start to final day of tumor measurement was assessed by comparing the geometric mean change in tumor volume (TV) for control and treated groups.

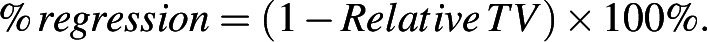


Percentage of TV change =  (*V*_end of study day_ – *V*_baseline_)/*V*_baseline_ ×100.

For biomarker analysis, tumors were harvested and snap-frozen in liquid nitrogen 4 or 24 hours after the last dose. Group sizes and treatments are described in corresponding figure legends. Significance was evaluated using a one-tailed *t* test compared with vehicle control on the day of final tumor measurement.

### Data availability

Raw sequencing data are available in ArrayExpress (accession number E-MTAB-13113 and E-MTAB-13139). All other raw data underlying this article's findings are available upon request from the corresponding author.

## Results

### Camizestrant potently inhibits proliferation in ER-driven breast cancer cell lines

**Figure 1.**
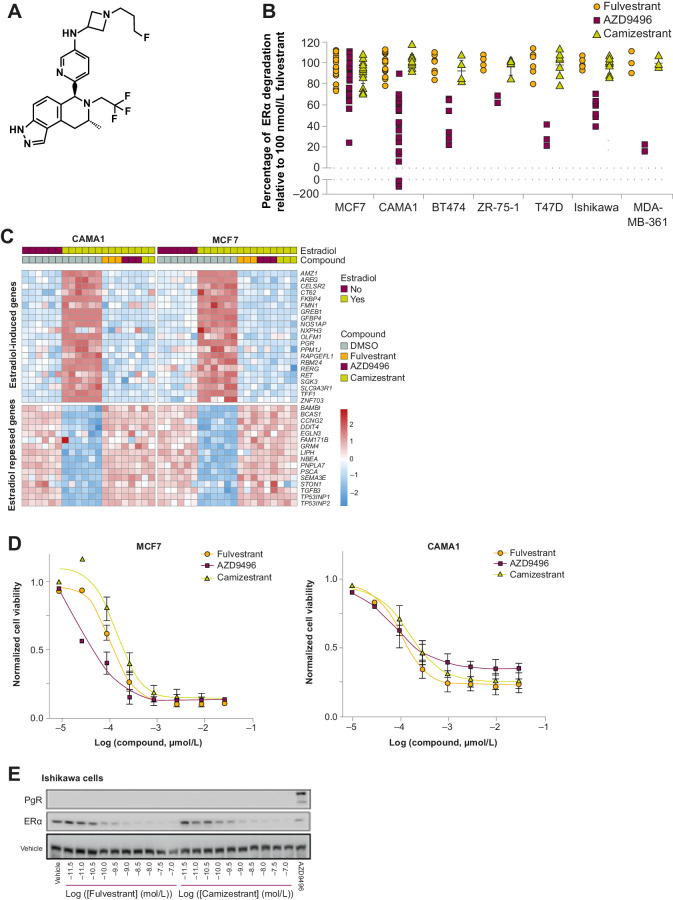
Camizestrant (AZD9833) is a selective ER degrader and pure antagonist. **A,** Chemical structure of camizestrant. **B,** The indicated cell lines were treated with 100 nmol/L of the indicated compound for 48 hours. Levels of ERα were assessed by Western blotting and normalized to an untreated control and fulvestrant. Each point represents an independent experiment. **C,** MCF7 or CAMA-1 cells were treated with DMSO, 1 nmol/L estradiol, or 100 nmol/L of the indicated compound + 1 nmol/L estradiol for 24 hours. RNA expression was assessed by RNA sequencing. Data represent z-scores of normalized gene expression for genes in an ER activity signature. **D,** MCF7 and CAMA-1 cells were treated with the indicated concentration of the indicated compound for 7 days. Cell number was estimated with a Sytox Green assay normalized to an untreated control on the day of treatment (0.0) and an untreated control on day 7 after treatment (1.0). Data points represent the mean from three independent experiments performed in triplicate ± SD. **E,** Ishikawa cells were treated with the indicated concentration of fulvestrant or camizestrant, or 100 nmol/L AZD9496 for 24 hours, and ERα and PgR expressions were determined by Western blot. DMSO, dimethyl sulfoxide; PR, progesterone receptor.

*In vitro*, camizestrant (Fig. 1A; ref. 35) degrades ER protein to the same extent as fulvestrant, and more effectively than the first-generation oral SERD AZD9496, across a panel of ER-driven cell lines (Fig. 1B; Supplementary Fig. S2A).

SERD-mediated ER degradation does not reduce ER levels completely, due to continuous *ESR1* expression and protein translation. We therefore quantified ER degradation over time, which showed that 100 nmol/L camizestrant increased the ER degradation rate, reducing the ER basal half-life from 2.54–3.86 to 0.46–0.70 hours (Supplementary Fig. S2B), as effectively as fulvestrant. Degradation was proteasome-mediated, as the proteosome inhibitor MG132 ablated protein reduction (Supplementary Fig. S2C). Subcellular fractionation revealed that following 100 nmol/L camizestrant or fulvestrant, all residual ER was within the chromatin fraction (Supplementary Fig. S2D).

To explore the consequence of different extents of ER degradation by camizestrant, fulvestrant, and AZD9496 in MCF7 and CAMA-1 cells, we evaluated ER transcriptional activity using RNA-seq gene expression. Following acute treatment, camizestrant, fulvestrant, and AZD9496 antagonized all estradiol-driven gene expression, consistent with complete antagonism of residual ER transcriptional activity (Fig. 1C). In 7-day proliferation experiments, camizestrant and fulvestrant inhibited cell growth equivalently in both cell lines, whereas AZD9496 displayed weaker antiproliferative effects in CAMA-1 cells (Fig. 1D).

Having no ER agonism is important for an ngSERD. Accordingly, we show that camizestrant degraded ER to the same extent as fulvestrant in the ER^+^ Ishikawa endometrial cancer cell line, an *in vitro* model of ER agonism (Fig. 1E), with no evidence of PgR expression (as a marker of agonism), in contrast with AZD9496. These data show that camizestrant promotes selective and proteasome-mediated ER degradation, completely suppresses transcription by fully antagonizing residual ER, and impairs proliferation in ER^+^ breast cancer cell lines, without agonizing ER in uterine models, in marked contrast with AZD9496.

#### Camizestrant inhibits proliferation in *ESR1*wt and *ESR1*m breast cancer models

**Figure 2.**
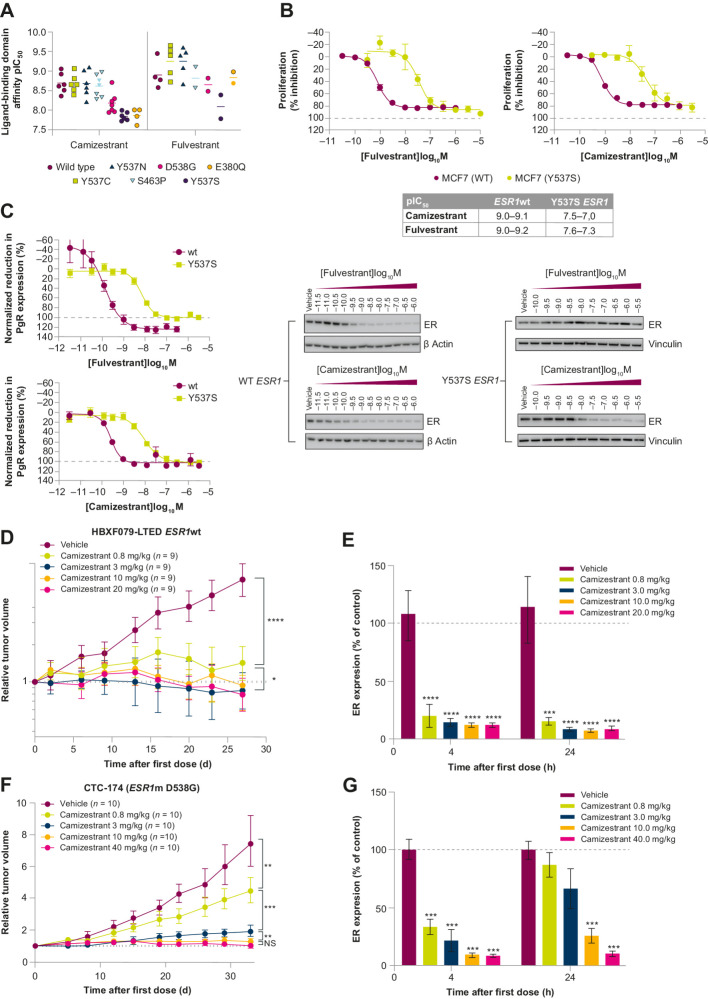
Binding and activity of camizestrant in clinically relevant ERα mutations. **A,** The pIC_50_ value of fulvestrant and camizestrant to displace a fluorescent ER ligand from wild-type, D538G, Y537N, E380Q, Y537C, S463P, or Y537S mutant purified ERα ligand-binding domain. Points represent independent experiments. **B,** MCF7 cells expressing WT or Y537S ERα were grown for 7 days in 5% FBS. Growth inhibition was estimated with a Sytox Green assay normalized to an untreated control on day 0 (0%) and an untreated control on day 7 of treatment (100%). Data points represent the mean from two independent experiments carried out in duplicate. Fulvestrant and camizestrant inhibited the proliferation of both WT and Y537S ERα-expressing MCF7 cells in a concentration-dependent manner. The table shows pIC_50_ values from independent experiments. **C,** MCF7 cells expressing WT or Y537S ERα were treated with the indicated concentration of fulvestrant or camizestrant for 72 hours, and ERα was determined by Western blot. Fulvestrant and camizestrant showed concentration-dependent inhibition of PgR expression (normalized to an untreated control) in MCF7 cells expressing both WT and Y537S ERα. **D,** Camizestrant dose–response in the long-term estrogen-deprived *ESR1*wt PDX model, HBXF079-LTED. Statistical analysis was performed by one-tailed, unequal variance *t* test versus log (change in tumor volume) compared with vehicle control at the final day of treatment. **E,** ER degradation measured by Western blot from tumors taken at the end of the efficacy dosing period. **F,** In the *ESR1*m D538G PDX CTC-174 model, camizestrant demonstrated antitumor activity in a dose-dependent manner, with maximal antitumor activity at 10 mg/kg. Efficacy correlated with ER degradation measured by Western blot from tumors taken at the end of the efficacy dosing period. Statistical analyses were performed by one-tailed, unequal variance *t* test versus log (change in tumor volume) compared with vehicle control at the final day of treatment. **G,** ER degradation measured by Western blot from tumors taken at the end of the efficacy dosing period. NS, not significant; *, *P* < 0.05; **, *P* < 0.01; ***, *P* < 0.001; ****, *P* < 0.0001. pIC_50_, negative log of the IC_50_ (half-maximal inhibitory concentration) value when converted to mol/L.

*ESR1* ligand-binding domain (LBD)–activating mutations are well-established mechanisms of resistance, detected in 20% to 40% of patients with metastases who progress after AI-containing regimens (36, 37). Therefore, we compared the binding potency of camizestrant with fulvestrant in several *ESR1*m variants. In competition binding assays, camizestrant bound with high affinity to the recombinant LBD of *ESR1* wild-type (*ESR1wt*) and *ESR1*m variants, including Y537C, Y537N, Y537S, S463P, D538G, and E380Q (Fig. 2A). Some loss of affinity was observed in binding to certain *ESR1*m, in particular Y537S for camizestrant and fulvestrant, and E380Q for camizestrant. Despite reduced binding, camizestrant and fulvestrant inhibited the growth of MCF7 cells expressing either wt or Y537S *ESR1* equivalently (Fig. 2B and C), and more potently than elacestrant (RAD1901; Supplementary Fig. S3A and S3B). These effects are as previously reported (35). The data suggest that despite reduced binding affinity compared with *ESR1*wt, camizestrant can inhibit proliferation of Y537S *ESR1*m cells.

In *ESR1*wt and *ESR1*m Y537S MCF7 cells, camizestrant and fulvestrant reduced ER and PgR levels more than did elacestrant (RAD1901; Supplementary Fig. S3A and S3B). Collectively, camizestrant's cellular activity matched fulvestrant's activity *in vitro*, and was positively differentiated from elacestrant.

In ER^+^ tumor models, camizestrant showed strong dose-dependent efficacy, with greater activity at lower doses in the *ESR1*wt HBXF079-LTED than in D538G *ESR1*m CTC174 PDX models (Fig. 2D–G); notably, camizestrant at 3 mg/kg daily still caused tumor stasis in the latter. Efficacy correlated with overall ER degradation levels, and similar dose-dependent efficacy occurred in MCF7 parental (35) and *ESR1*m Y537S xenograft models (Supplementary Figs. S3C and S3D and S4). The data indicate a similarly reduced potency in *ESR1*m versus *ESR1*wt models (as in protein binding and cell lines), but nevertheless suggest that camizestrant still achieves high ER reduction and efficacy at relevant doses.

Next, we established a pharmacokinetic/pharmacodynamic mathematical model to assess tumor ER levels during camizestrant treatment in the CTC174 hormone-independent PDX model (Supplementary Fig. S4). Camizestrant-free plasma concentration clearly correlated with ER levels, with an estimated *in vivo* free IC_50_ value of 0.4 nmol/L (Supplementary Fig. S4A). The magnitude and duration of ER degradation with daily camizestrant was clearly dose-responsive (Supplementary Fig. S4B). Moreover, the association between ER degradation during the dosing interval and camizestrant's antitumor effect showed that maximal antitumor effects occur with 10 mg/kg once-daily dosing, achieving an average 87% ER degradation (i.e., only 13% ER remaining; Supplementary Fig. S4C). This demonstrates a link between camizestrant exposure, ER degradation, and antitumor effects *in vivo*, with associated antagonism of remaining ER in line with the *in vitro* data.

Having established camizestrant 10 mg/kg daily as the efficacious dose, we confirmed *in vitro* results, showing complete ER antagonism in Ishikawa cells (Supplementary Fig. S5A). A 10 mg/kg once-daily dose in juvenile rats slightly reduced uterine weight and endometrial epithelium area, consistent with the lack of agonism seen *in vitro*, and contrasting with the effects of partial ER agonists tamoxifen and AZD9496 (Supplementary Fig. S5B–S5D).

#### Camizestrant delivers broader activity than fulvestrant in *ESR1*wt and *ESR1*m PDXs by enhancing suppression of ERα and cell-cycle genes

**Figure 3.**
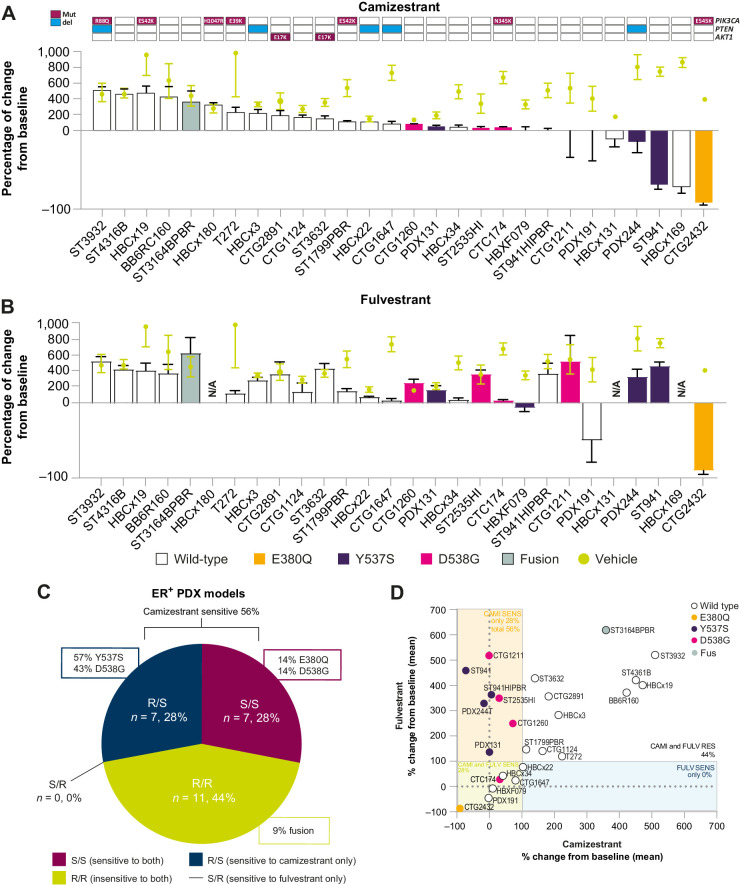
Camizestrant has superior *in vivo* activity to fulvestrant in *ESR1*wt and *ESR1*m PDX models (1). **A,** Waterfall plot representing the growth of 28 of ER^+^ breast cancer PDX treated with camizestrant 10 mg/kg daily; bars are colored according to *ESR1* mutational status and other genomic characteristics annotated at the top. The percentage change calculated from the initial volume at day of treatment is shown. Data represent mean ± SE of the mean. **B,** Waterfall plot representing the growth of 25 PDX treated with fulvestrant 5 mg/kg weekly. N/A denotes models where the head-to-head arm was not available. The percentage of change calculated from the initial volume at day of treatment is shown. Data represent mean ± SE of the mean. **C,** Pie chart indicates the proportions of PDX models sensitive or insensitive to camizestrant and/or fulvestrant. The antitumor response of camizestrant versus fulvestrant monotherapy is represented as the percentage of tumor change compared with the initial tumor volume, benchmarked to vehicle changes. **D,** Correlation of fulvestrant PDX antitumor response (*y*-axis) versus camizestrant (*x*-axis) in 25 PDX, represented as the percentage of tumor volume change compared with the initial tumor volume. Data represent mean. The boxes indicate the percentage change from baseline ≤100%; the percentage of models sensitive only to fulvestrant or camizestrant, or sensitive to both is represented in each box.

Camizestrant delivers superior ERα antagonism and signaling inhibition than fulvestrant. Therefore, we compared the activity of camizestrant with fulvestrant in *in vivo* ER^+^ PDX tumor models representing various disease stages and genomic alterations (Fig. 3; Supplementary Fig. S6).

Camizestrant 10 mg/kg daily was profiled in 28 PDX models, and head-to-head with fulvestrant at 5 mg/weekly in 25 ER^+^ PDX models derived from primary or metastatic tumors, naïve or exposed to ET and/or CDK4/6i, and with various *ESR1*m status (Fig. 3A and B). Camizestrant showed antitumor activity in twice as many models as fulvestrant, with efficacy in 56% of *ESR1*wt and *ESR1*m models, with fulvestrant effective in 28% (Fig. 3C). Some (44%) models were insensitive to both compounds, suggesting absent ER-drive in those tumors. Remarkably, no model was fulvestrant-sensitive but camizestrant-insensitive (Fig. 3C and D). When overlaying *ESR1*m status on the analysis, 36% of models had *ESR1*m, including Y537S, D538G, and E380Q. Camizestrant was active, and fulvestrant inactive, in all Y537S models tested (Fig. 3C and D). This comprehensive set of PDX models shows that camizestrant delivers superior efficacy over fulvestrant, at clinically achievable doses, in *ESR1*m and *ESR1*wt models from primary and metastatic tumors.

Given the striking superiority of camizestrant in these PDX models, we analyzed a subset of them, classifying their genetic features and biomarker modulation compared with fulvestrant. We extended the efficacy comparison with elacestrant 30 mg/kg daily in three models. We observed equivalent activity between camizestrant and fulvestrant in three models [two *ESR1*wt (HBXF-079 and PDX191) and one *ESR1*m D538G (CTC174); Supplementary Fig. S7A, S7B, and S7E], and superior activity for camizestrant in four *ESR1*m models (PDX244, ST941/HI, PDX131, Y537S, and a D538G *ESR1*m CTG1211; Supplementary Fig. S77C, S7D, S7F, and S7G). In the PDX191 model, although a trend toward sensitivity to fulvestrant was observed, this did not reach statistical significance. In Y537S *ESR1*m PDX ST941/HI and PDX244 models, camizestrant promoted more profound and sustained efficacy than elacestrant, whereas both delivered similar efficacy in the HBXF-079 model (Supplementary Fig. S7E–S7G). These data support a superior efficacy profile of camizestrant versus both fulvestrant and elacestrant in Y537S PDX models.

End-of-study tumor ER protein levels were measured to understand whether the differential efficacy of camizestrant, fulvestrant, and elacestrant was due to the extent of protein degradation *in vivo.* Although in the CTC174 model—equally sensitive to camizestrant and fulvestrant—we observed similar ER degradation levels, in the ST941/HI model (camizestrant-sensitive/fulvestrant-insensitive), elacestrant degraded ER less than fulvestrant and camizestrant 4 and 24 hours after treatment (Supplementary Fig. S6H). Therefore, in these models the difference in activity cannot result solely from ER degradation differences.

**Figure 4.**
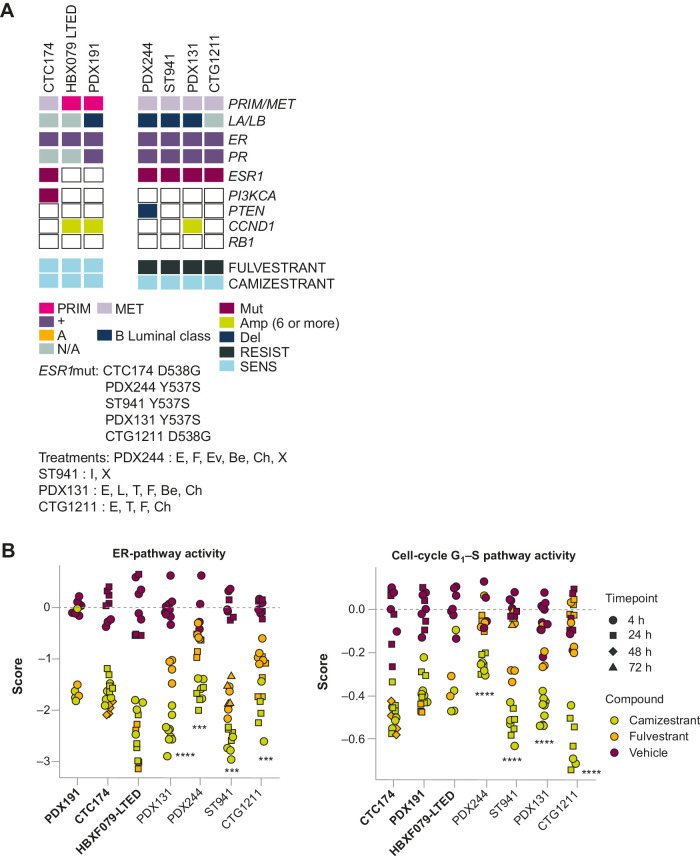
Camizestrant has superior *in vivo* activity to fulvestrant in *ESR1*wt and *ESR1*m PDX models (2). **A,** Characteristics of ER^+^ breast cancer models used. **B,** Change in ER pathway gene activation after treatment, expressed as change in ER pathway gene score and in cell-cycle G_1_–S checkpoint genes. See Supplementary Methods for details. Statistical analysis comparing fulvestrant and camizestrant was done using one-way analysis of covariance (*n* ≥ 4 animals per group). Models shown in bold (*x*-axis) are fulvestrant sensitive; those in regular type are fulvestrant resistant. ***, *P* < 0.001; ****, *P* < 0.0001. Amp, amplification; CCND1, cyclin D1; Del, deletion; MET, metastasis; Mut, mutation; PIK3CA, phosphatidylinositol 3-kinase subunit α; PR, progesterone receptor; PRIM, primary; RESIST, resistant; RB1, retinoblastoma gene; SENS, sensitive. Patient treatment reported: Be, bevacizumab; Ch, chemotherapy; E, exemestane; Ev, everolimus; F, fulvestrant; I, investigational; L, letrozole, T, tamoxifen; X, radiotherapy.

To understand the mechanism allowing camizestrant to overcome fulvestrant resistance, we measured, using genomic characterization and RNA-seq analysis, non-genomic alterations and ER pathway transcriptional output under drug treatment in seven PDX models. The models were characterized by treatment response, *ESR1*m status, and other molecular features (Fig. 4A). We observed clear enrichment of patient-derived metastatic tumors harboring *ESR1*m in the four PDX models unresponsive to fulvestrant but sensitive to camizestrant.

Gene expression analysis comparing the four fulvestrant-resistant PDX models with the three fulvestrant-sensitive PDX models showed that camizestrant more strongly modulated gene signatures than fulvestrant, as demonstrated by both ER (Fig. 4B) and estradiol gene signature scores (defined by expression of estradiol-induced or estradiol-repressed genes; Supplementary Fig. S7I), which correlated with camizestrant efficacy. Therefore, camizestrant treatment in fulvestrant-resistant models more strongly antagonizes ER and changes the transcriptional signature, including superior suppression of the ER pathway, genes defining early or late response to estrogen, and downstream cell-cycle–related gene signatures (Fig. 4B; Supplementary Fig. S7I).

#### Camizestrant improves tumor control in D538G *ESR1*m and N354K *PIK3CA*m PDX CTC174 in combination with CDK4/6 or PI3K/AKT/mTOR inhibitors *ex vivo* and *in vivo*

**Figure 5.**
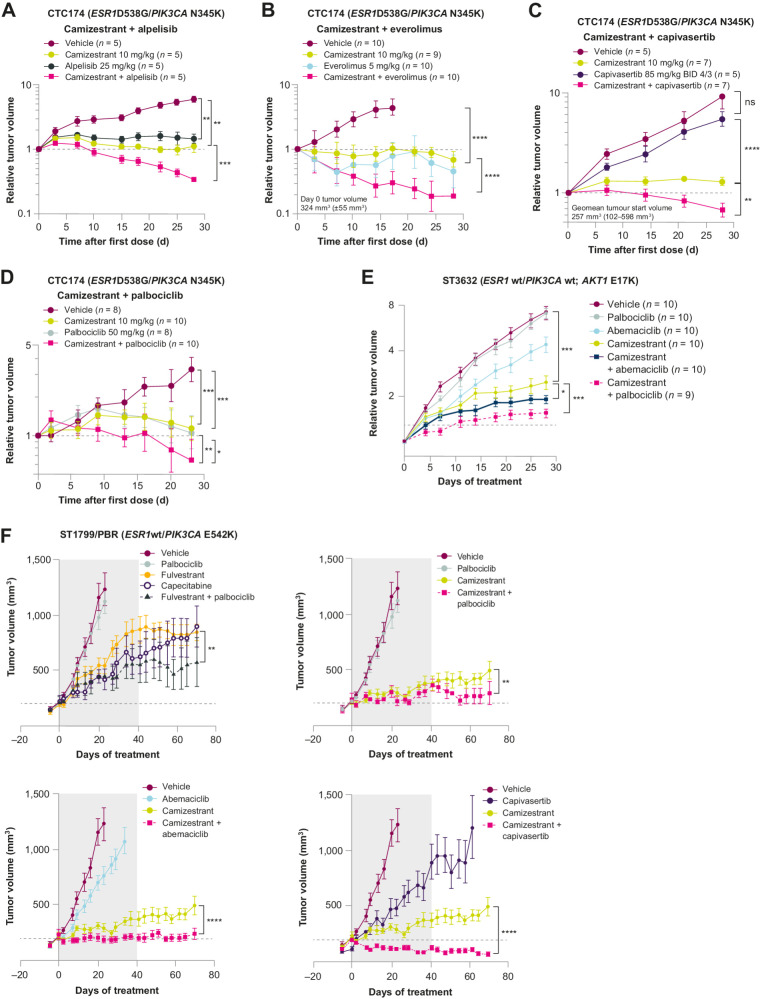
Enhanced efficacy of camizestrant in combination with PI3K/AKT/mTOR inhibitors as doublets in CDK4/6-sensitive and -resistant models (1). **A–D,** Combination of camizestrant with PI3Kα inhibitor alpelisib (**A**), mTOR inhibitor everolimus (**B**), AKT inhibitor capivasertib (**C**), or CDK4/6 inhibitor palbociclib (**D**) delivers enhanced efficacy compared with monotherapy in D538G *ESR1*m PDX CTC-174. Statistical analysis was performed by one-tailed, unequal variance *t* test versus log (change in tumor volume) compared with vehicle control at the final day of treatment. **E,** Relative tumor volume plots of ST3632 PDX model treated with oral camizestrant at 10 mg/kg daily, oral palbociclib at 50 mg/kg daily, and oral abemaciclib 50 mg/kg daily, and with camizestrant + abemaciclib and camizestrant + palbociclib. Statistical analysis was performed by one-tailed, unequal variance *t* test versus log (change in tumor volume) at the final day of treatment. **F,***In vivo* combination of camizestrant at 10 mg/kg daily with palbociclib 50 mg/kg, abemaciclib 50 mg/kg, and capivasertib 130 mg/kg in PDX ST1799 dosed for 40 days (gray area). For clarity, the graph is divided into four subgraphs due to the large number of treatment arms; where they appear, the vehicle, camizestrant, and palbociclib arms are the same in each subgraph. CDK, cyclin-dependent kinase. *, *P* < 0.05; **, *P* < 0.01; ***, *P* < 0.001; ****, *P* < 0.0001; ns, not significant.

**Figure 6.**
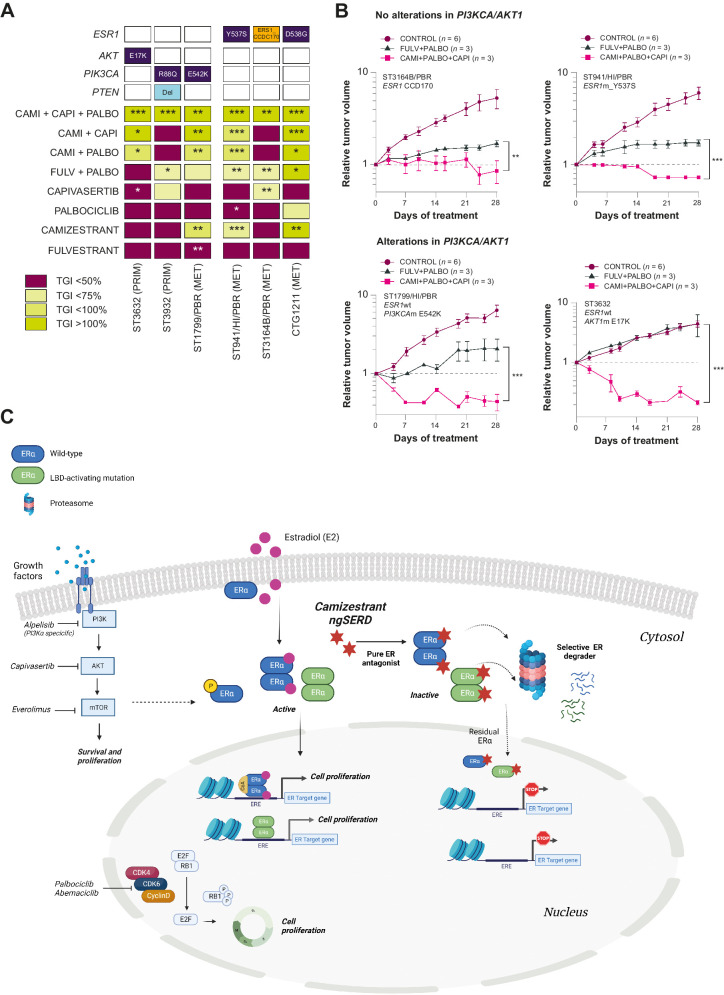
Enhanced efficacy of camizestrant in combination with PI3K/AKT/mTOR inhibitors as doublets in CDK4/6i-resistant models (2). **A,** 28-, 35- or 42-day efficacy studies using several ER^+^ breast cancer PDX harboring/not harboring alterations in *PIK3CA/AKT/PTEN*. Dark blue, mutations; light blue, deletions; orange, fusions. The rate of growth for each animal is estimated on the basis of fitting each tumor's growth curve to an exponential model: log_10_(tumor volume) = *a* + *b*·time + error, where *a* and *b* correspond to the log initial volume and growth rate, respectively. The model assumes that the error terms are normally distributed. Tumor volumes less than 15 mm^3^ were replaced with a minimum value of 15 mm^3^. This growth rate summary metric was then used for statistical analysis to compare treatments with a user-specified reference group. Tumor growth inhibition was used to plot a heat map. Designed dosing: oral palbociclib 50 mg/kg daily, subcutaneous fulvestrant 5 mg weekly, oral camizestrant 10 mg/kg daily, oral capivasertib 130 mg/kg BID 4 days on/3 days off. Statistical analysis was performed by one-tailed, unequal variance *t* test versus log (change in tumor volume) at the final day of treatment. **B,** 28-, 35- or 42-day efficacy studies used in **A**; relative tumor volume plots displaying arms: control, standard-of-care hormone therapy + CDK4/6 inhibitor (fulvestrant + palbociclib), or triplet combination of hormone therapy + CDK4/6 inhibitor + AKTi (camizestrant + palbociclib + capivasertib). Designed dosing: oral palbociclib 50 mg/kg daily, subcutaneous fulvestrant 5 mg weekly, oral camizestrant 10 mg/kg daily, oral capivasertib 130 mg/kg BID 4 days on/3 days off. Statistical analysis was performed by one-tailed, unequal variance *t* test versus log (change in tumor volume) at the final day of treatment. **C,** Camizestrant fits centrally in the overall landscape of breast cancer as a backbone endocrine therapy. Estrogens (e.g., E2) bind to ERα, leading to its dimerization and translocation to the nucleus, where ERα dimers bind to coactivators to form transcriptionally active ERα complexes. Activated complexes regulate gene transcription in the nucleus or activate kinases in the cytoplasm to drive cell proliferation. Mutations in the ligand-binding domain of *ESR1* drive resistance in advanced ER^+^ breast cancer and act independently of estrogens to activate transcription. Camizestrant is a next-generation SERD for the treatment of ER^+^ breast cancer, acting as a pure ER antagonist and selective ERα degrader. Camizestrant's mechanism of action stops the transcription of ER target genes in wild-type (blue) and mutant (green) ERα, impairing tumor cell proliferation. These properties position camizestrant as a central endocrine therapy partner along with CDK4/6 inhibitors (palbociclib and abemaciclib) in ER^+^ breast cancer. Other signaling pathways are essential to ER^+^ breast cancer proliferation and survival, and contribute to mechanisms of endocrine therapy resistance, including CDK4/6 and PI3K/AKT/mTOR pathways. Inhibitors of these signaling axes are currently approved targeted therapies (everolimus and alpelisib) or under investigation (e.g., capivasertib). *, *P* < 0.05; **, *P* < 0.005; ***, *P* < 0.0005. CAMI, camizestrant; CAPI, capivasertib; CDK, cyclin-dependent kinase; CoA, cytochrome C oxidase assembly; Del, deletion; E2, estradiol; E2F, E2F transcription factor; ERE, estrogen response element; FULV, fulvestrant; m, mutated; MET, metastatic; PALBO, palbociclib; PRIM, primary; RB1, retinoblastoma gene; TGI, tumor growth inhibition.

Given the high activity of camizestrant monotherapy in ER^+^ PDX models, we investigated camizestrant as the central ET partner to CDK4/6i and other standard-of-care therapies in ER^+^ breast cancer. Alpelisib (PI3Kα inhibitor) and everolimus (mTOR inhibitor) plus ET are approved in biomarker-selected PI3K/PTEN/AKT-altered populations and late-therapy lines, respectively (38). Therefore, the combinatorial potential of camizestrant with mTOR, AKT, PI3K or CDK4/6 inhibitors was tested *ex vivo* and *in vivo* (Figs. 5 and 6).

Experiments were conducted in PDX CTC174, a D538G *ESR1*m, and N354K *PIK3CA*m models. First, viability assays were performed in *ex vivo* PDX-derived organoids from CTC174 tumors. Camizestrant added benefit when combined with agents targeting the PI3K/AKT/mTOR pathway, alpelisib, everolimus, capivasertib (AKT inhibitor, AKTi), and palbociclib (CDK4/6i; Supplementary Fig. S8A and S8B). Next, we validated the benefits of these combinations *in vivo* (Fig. 5A–D). All combinations were more effective than monotherapy, with protein analysis from one study demonstrating downregulation of ER, the PI3K/AKT/mTOR pathway pS6/S6, or proliferation (proliferating cell nuclear antigen, PCNA) biomarkers after camizestrant, alpelisib, or combination treatment (Supplementary Fig. S8C).

#### Camizestrant is active in models resistant to CDK4/6i *in vitro* and *in vivo*

Effective ways to treat resistance to ETs and CDK4/6i is a high unmet need in ER^+^ breast cancer. Our experiments demonstrate that in CTC174, a model sensitive to ET and CDK4/6 inhibition, camizestrant and palbociclib showed a combination benefit. Therefore, we next investigated camizestrant combinations to overcome palbociclib (CDK4/6i) resistance in preclinical models. Camizestrant monotherapy efficacy was analyzed in MCF7 palbociclib-resistant cell lines (PC1, PC6, and PC8), which acquired resistance to palbociclib via different cellular mechanisms, including loss of RB1, cyclin D1, and *CCNE* (cyclin E) overexpression, and unknown mechanisms (Supplementary Fig. S8D). Palbociclib-resistant lines were sensitive to camizestrant, with no added benefit observed when combining with palbociclib (Supplementary Fig. S8D). However, palbociclib-resistant lines were sensitive to an alternative CDK4/6i, abemaciclib, with a broader CDK selectivity profile (39). Single-agent abemaciclib efficacy was enhanced when combined with camizestrant in palbociclib-resistant lines, supporting the rationale of treatment with alternative combinations of ET and CDK4/6i to overcome acquired resistance.

Next, we evaluated camizestrant efficacy in the PDX model ST1799/HI/PBR, which represents acquired palbociclib resistance and partial sensitivity to fulvestrant or the combination. In this *ESR1*wt model, prolonged camizestrant exposure decreased tumor growth compared with fulvestrant (Fig. 5F), and combined camizestrant and either palbociclib or abemaciclib further decreased TV (vs. fulvestrant and palbociclib combined). Efficacy was even sustained for approximately 30 days after treatment withdrawal. Although the ST1799/PBR model has an activating E542K *PIK3CA* mutation, capivasertib (AKTi) monotherapy could not completely arrest tumor growth. However, capivasertib combined with camizestrant caused tumor regressions that were sustained even after drug withdrawal. The data support combined approaches with camizestrant plus palbociclib, abemaciclib, or capivasertib in patients who progress on CDK4/6i treatment.

Therapies for early-stage ER^+^ disease to extend relapse-free survival are an unmet need, and oral SERDs are starting to be tested in the adjuvant setting. Therefore, we evaluated camizestrant in ST3632, an *ESR1*wt PDX model derived from a primary tumor of a treatment-naïve patient, reflecting early-stage disease. Camizestrant monotherapy reduced relative TV by 75% compared with the vehicle, showing greater efficacy than abemaciclib (46% inhibition) or palbociclib (0%). Combining camizestrant with either palbociclib or abemaciclib reduced TV further (91%; Fig. 5E), which corresponded with decreased ER and PgR proteins (Supplementary Fig. S8E). These results suggest that camizestrant alone or combined with CDK4/6i could be efficacious in early disease.

#### Combining camizestrant with CDK4/6i and AKTi inhibits tumor growth in models that progress on CDK4/6i, regardless of genetic alterations in *ESR1/PIK3CA/AKT/PTEN*

Data suggest that cotargeting CDK4/6, PI3K/AKT/mTOR, and ERα could effectively control tumor growth in ER^+^ breast cancer xenografts and PDX models (14, 40). Earlier, we showed camizestrant has antitumor activity in twice as many ER^+^ breast cancer PDX models as fulvestrant, including those derived from tumors that are primary or metastatic, naïve or exposed to ET and/or CDK4/6i, and with different *ESR1*m status (Figs. 2–4). Therefore, we tested camizestrant as the ET backbone in a triple combination with CDK4/6i and agents targeting the PI3K/AKT/mTOR pathway. We compared six PDX models representing CDK4/6i resistance via intrinsic mechanisms or induced by long-term palbociclib treatment. Models were classified on the basis of *ESR1*m or *PIK3CA/AKT1/PTEN* genetic alteration. The percentage of tumor growth inhibition of single, dual, and triple combinations relative to control vehicle was determined (Fig. 6A). Although camizestrant and capivasertib (individually and combined) at clinically relevant doses and schedules were active in most models, the camizestrant, palbociclib, and capivasertib triplet induced greater antitumor activity, regressing all models irrespective of genomic alterations, including *PTEN* loss, *PIK3CA*m, *ESR1*m, and an *ESR1*–*CCDC170* oncogenic fusion (Fig. 6A and B, Supplementary Fig. S1; refs. 41, 42). End-of-study protein samples from the BB6RC160 model (*ESR1*wt, no *PIK3CA/PTEN/AKT1* alterations) were collected after 21 days’ treatment to assess pathway modulation (Supplementary Fig. S1C). Protein analysis demonstrated significant modulation of ER (ER, TFF1), proliferation (PCNA), CDK4/6 (pRb1, Rb1), and PI3K/AKT/mTOR signaling proteins (pAKT, pPRAS40, pS6 and total) after triplet treatment (Supplementary Fig. S1C). The data suggest that co-targeting these three signaling nodes to inhibit ER, CDK4/6, and AKT with camizestrant as an ET backbone could expand activity in patients with refractory tumors.

## Discussion

Using various *in vitro* and *in vivo* assays, we demonstrate that monotherapy with the ngSERD camizestrant robustly and selectively degrades ER, with pure ER antagonism. This translates into antitumor activity in models representing the clinical landscape in ER^+^ breast cancer (Fig. 6C). Notably, camizestrant showed *in vivo* activity in *ESR1*wt models and in models bearing clinically relevant *ESR1*m, such as D538G and Y537S: This has significant implications for patients whose disease acquires resistance to standard ET. Furthermore, camizestrant arrested or regressed tumor growth in *ESR1*m PDX models in which fulvestrant had poor or no activity, suggesting that camizestrant may also benefit patients who do not benefit from fulvestrant, a current ET standard of care.

Consistent with our data, others have suggested that SERDs can differ in their ability to degrade ER in ER^+^ breast cancer cell lines, and that this correlates with a partial agonist phenotype and poorer anti-proliferative activity in the cell lines where degradation is compromised (43). Specifically, SERDs with acidic headgroups, such as AZD9496 and GDC-0810, substantially degrade ER in only some cell lines, whereas SERDs with basic headgroups, such as camizestrant and GDC-0927, phenocopy fulvestrant and have substantially degraded ER in all cell lines tested.

Interestingly, this differing ability of acidic and basic SERDs to degrade ER resulted in no differential effect on ER-regulated gene expression in CAMA-1 cells. Our data on the antiproliferative effects of acidic and basic SERDs align with a recent report (44), in which subtle differences in the antiproliferative effects of GDC-0810 and GDC-0927 are described previously.

Partial ER agonism is an undesirable side effect of tamoxifen, increasing the risk of endometrial cancer (45). With the acidic SERD AZD9496, agonism was undetected in ER^+^ breast cancer cell lines, but evident in endometrial *in vitro* and *in vivo* models, which are more sensitive for detecting ER agonism. This aligns with data we (23) and others (44, 46) have published. The SERM/SERD elacestrant does not degrade ER to the same extent as fulvestrant in multiple ER^+^ breast cancer cell lines, and agonizes ER in mouse uterine tissue (47). Contrastingly, even in these more sensitive systems, agonism was not seen with the basic ngSERD camizestrant *in vitro* or *in vivo.*

The precise mechanism by which SERDs degrade ER is unclear, as is why the degree of ER degradation differs among SERDs and cell lines. Although the 26S proteasome inhibitor MG132 can prevent SERD-induced ER degradation, to date, no specific E3-ligase has been found to be involved. Furthermore, fulvestrant-like SERDs immobilize ER on chromatin more than acidic SERDs (43), but in MCF7 cells both SERD types degrade ER equivalently. Therefore, the molecular mechanism behind this difference remains elusive.

Among our most notable findings is camizestrant's improved antitumor activity and ER pathway modulation in *ESR1*m PDX models compared with both fulvestrant and elacestrant. This occurs despite fulvestrant doses in mouse studies exceeding clinical exposures approximately 8-fold, likely overestimating fulvestrant's clinical potential (48). These data align with the observation from the PALOMA-3 trial that Y537S *ESR1*m—but not other *ESR1*m variants—was enriched in fulvestrant-treated patients (with or without palbociclib) at progression. This suggests that Y537S mutations can cause fulvestrant resistance (37). Conversely, camizestrant has demonstrated clinical activity in patients with HR^+^/HER2^−^ metastatic breast cancer with a variety of *ESR1*m, including Y537S, and reduces Y537S *ESR1*m levels in circulating tumor DNA (49). This difference in activity of camizestrant and fulvestrant in patients with detectable *ESR1*m and *ESR1*wt is being explored in the ongoing Phase 2 SERENA-2 trial (NCT04214288), comparing the efficacy of camizestrant versus fulvestrant in women with ER^+^/HER2^–^ advanced breast cancer. SERENA-2 has recently reported positive results; notably, camizestrant improves median PFS compared with fulvestrant in patients with detectable *ESR1*m at baseline, including the more commonly detected D538G and Y537C/D/N/S *ESR1*m variants (50). This suggests that camizestrant may benefit those with ET resistance.

Importantly, camizestrant showed improved activity compared with elacestrant in a subset of preclinical models. Elacestrant has demonstrated modest clinical improvement in median PFS over fulvestrant and AIs in ER^+^/HER2^−^ advanced breast cancer following treatment with CDK4/6i (EMERALD; ref. 29). We also showed that in models sensitive to camizestrant but insensitive to fulvestrant, only camizestrant can sufficiently block the ER and cell-cycle pathways at the transcriptional level, correlating with the observed efficacy. This effect could be due to a more complete antagonism of residual-free or chromatin-bound ER compared with fulvestrant, but further experiments are warranted to understand the precise mechanisms. The data presented suggest putative mechanisms by which camizestrant has superior activity compared with fulvestrant.

Camizestrant exhibited favorable efficacy *in vivo* as monotherapy and in combination with CDK4/6 or PI3K/AKT/mTOR inhibitors. Combination treatments inhibited tumor growth more than did single treatments in *ESR1*m and *PIK3CA*m PDX models. This increase in activity was also observed in ER^+^ PDX models insensitive to palbociclib and/or ET. These data support the benefit of camizestrant and PI3K/AKT/mTOR inhibitors in patients with *ESR1*m and *PIK3CA*m tumors.

Molecular mechanisms of resistance to ET plus CDK4/6i are incompletely understood, and novel approaches for patients who relapse after treatment are a high unmet need (12). Our results support camizestrant's potential to be a superior backbone ET in naïve, early-stage disease, as well as in late-stage ER^+^/HER2^−^ breast cancer tumors that have progressed on current ETs with/without CDK4/6i. Moreover, our data suggest that a novel triple combination of camizestrant, capivasertib, and CDK4/6i could help to achieve high response rates in a broader patient population, irrespective of *ESR1*m and *PIK3CA/AKT1/mTOR* alterations, in the metastatic and CDK4/6i-resistant setting. Although all six preclinical models tested benefited from a triplet combination, further studies can stratify biomarkers to select appropriate combinations for specific breast cancer populations.

On the basis of these preclinical data, clinical investigation of camizestrant is underway in Phase 2 and 3 trials. In addition to SERENA-2, SERENA-4 (NCT04711252) is comparing camizestrant and palbociclib versus anastrozole and palbociclib as first-line treatment for women with ER^+^/HER2^–^ advanced breast cancer. SERENA-6 (NCT04964934) is comparing the effects of switching to camizestrant in combination with palbociclib or abemaciclib versus continuing anastrozole or letrozole in combination with palbociclib or abemaciclib in patients with ER^+^/HER2^−^ metastatic breast cancer with detectable *ESR1*m, who are already receiving first-line treatment (51). In addition to these studies in advanced disease, CAMBRIA-1 (NCT05774951) is an open-label Phase 3 study evaluating outcomes with camizestrant versus standard ET for patients with ER^+^/HER2^−^ early breast cancer and at least 2 years of standard adjuvant ET. These studies contribute to camizestrant's comprehensive development program.

In summary, we provide evidence supporting the clinical development of the ngSERD camizestrant as monotherapy, or in double or triple combinations with CDK4/6 and AKT inhibitors, to block ER signaling more completely than currently available ETs for patients with ER^+^ breast cancer, and improve therapeutic outcomes.

### Conclusions

Building on the success of fulvestrant, we show that the ngSERD camizestrant could become the next-generation ET for patients with HR^+^ breast cancer across the clinical landscape. Compared with fulvestrant, camizestrant shows enhanced efficacy and superior ER signaling and cell-cycle suppression in PDX models. Clinically, camizestrant could enhance ER antagonism and degradation, thereby improving clinical benefit for patients with HR^+^ breast cancer. Camizestrant also has potential, in combination with CDK4/6 and PI3K/AKT/mTOR inhibitors, to address resistance to current ETs: The highest unmet need in the largest group of patients with breast cancer.

## Supplementary Material

Supplementary Data Supplementary file containing all supplementary tables, text and figures
