# Supplementary material for: The Next-Generation Oral Selective Estrogen Receptor Degrader Camizestrant (AZD9833) Suppresses ER+ Breast Cancer Growth and Overcomes Endocrine and CDK4/6 Inhibitor Resistance
Source: Cancer Res. 2023 Sep 19;83(23):3989–4004. doi: 10.1158/0008-5472.CAN-23-0694 (PMC10690091; doi:10.1158/0008-5472.CAN-23-0694)
Supplement: Supplementary Data — Supplementary file containing all supplementary tables, text and figures [file can-23-0694_supplementary_data_suppsf1-sf8stxt.pdf]

The next-generation oral selective estrogen receptor degrader camizestrant (AZD9833) suppresses ER+ breast cancer growth and overcomes endocrine and CDK4/6 inhibitor resistance

Supplementary data

Supplementary Figure 1. Efficacy of mono- and combination therapy in PIK3CA AKT-*altered and non-altered models*

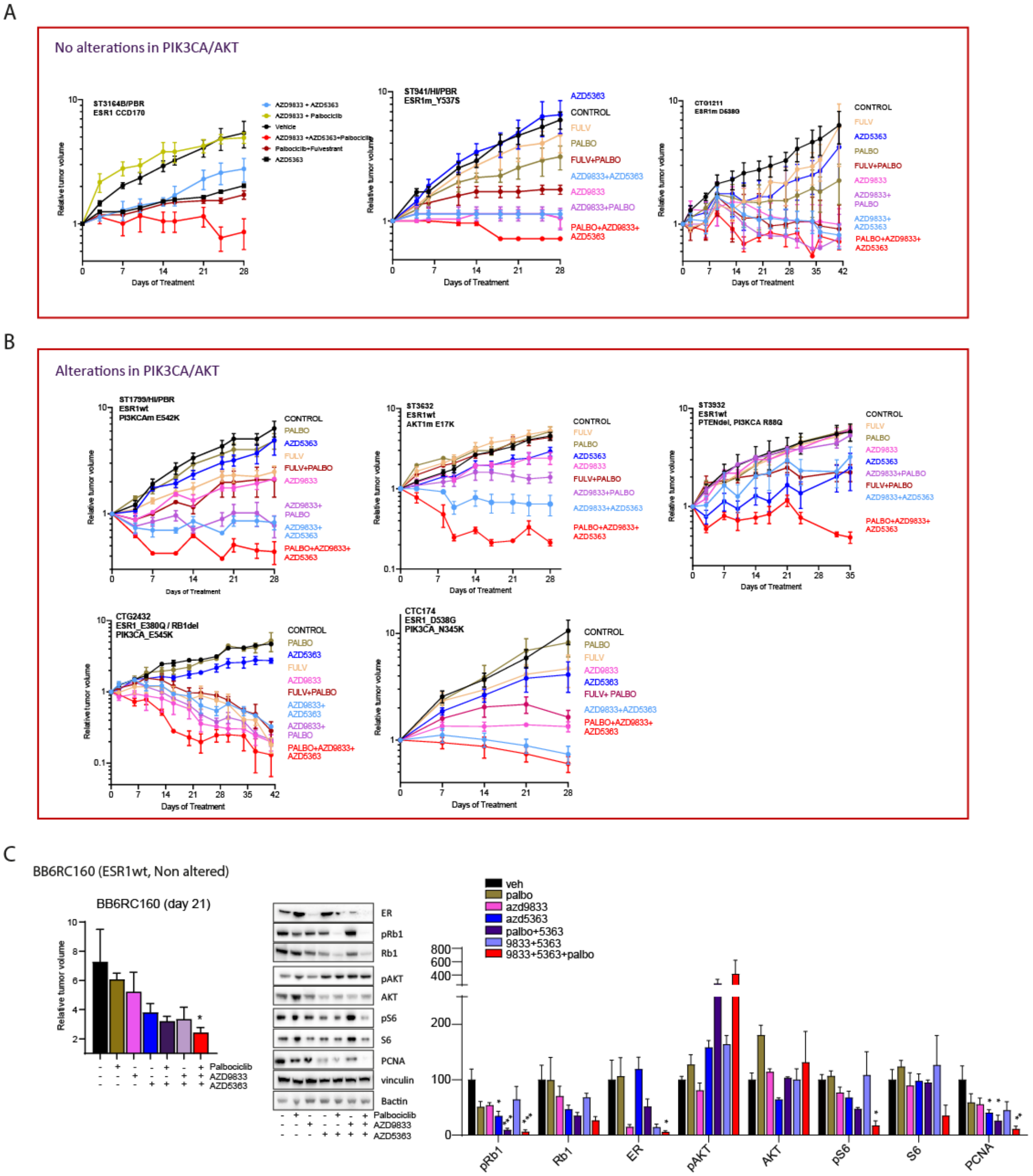

A) 28- or 42-day efficacy studies, relative tumor volume plots for non-altered *PIK3CA/AKT* or *PTEN* ER+ breast cancer PDX, displaying all treatment arms. Designed dosing: oral palbociclib 50 mg/kg four times daily, subcutaneous fulvestrant 5 mg/kg weekly, oral camizestrant 10 mg/kg four times daily, capivasertib (shown in figure as AZD5363) 130 mg/kg twice daily x 4 x 11. Some arms are shared with B) (i.e. control, fulvestrant + palbociclib, and camizestrant + palbociclib + capivasertib). **B)** 28-, 35- or 42-day efficacy studies used in A); relative tumor volume plots for altered *PIK3CA/AKT* or *PTEN* ER+ breast cancer PDX, displaying all treatment arms. Designed dosing: oral palbociclib 50 mg/kg (except for CTC174 used at 25 mg/kg) daily; subcutaneous fulvestrant 5 mg/kg weekly; oral camizestrant 10 mg/kg four times daily; capivasertib 130 mg/kg (except for CTC174 used at 85 mg/kg) twice daily x 4 x 11. **C)** Left: 21-day efficacy using BB6R160 ER+ PDX (*ESR1*wt, no alterations *PIK3CA/AKT* or *PTEN*), displaying all treatment arms at end of study (n=3 animals per group) with camizestrant 10 mg/kg daily, palbociclib 25 mg/kg daily, AZD5363 100 mg/kg 4- on 3-off, and combinationl treatments; Middle: End-of-study samples were used to analyze protein content after treatment, representative western blot; Right: quantification of protein detected using end-of-study samples. Statistical analysis was performed by ANOVA. *p<0.05; **p<0.01; ***p<0.001.

AKT, Akt serine/threonine kinase; ANOVA, analysis of variance; Cami/AZD9833, camizestrant; Capi/AZD5363, capivasertib; CDK4/6(i), cyclin-dependent kinase 4/6 (inhibitor); Ctrl, control; ER, estrogen receptor; *ESR1*, estrogen receptor 1 gene; Fulv, fulvestrant; p, phosphorylated; Palbo, palbociclib; PDX, patient-derived xenograft; PCNA, proliferating cell nuclear antigen; PIK3CA, phosphatidylinositol 3-kinase subunit α; Rb, retinoblastoma; S6, Ribosomal protein S6; veh, vehicle; wt, wild-type.

Supplementary Figure 2. Camizestrant induces proteasome-mediated degradation of ERα

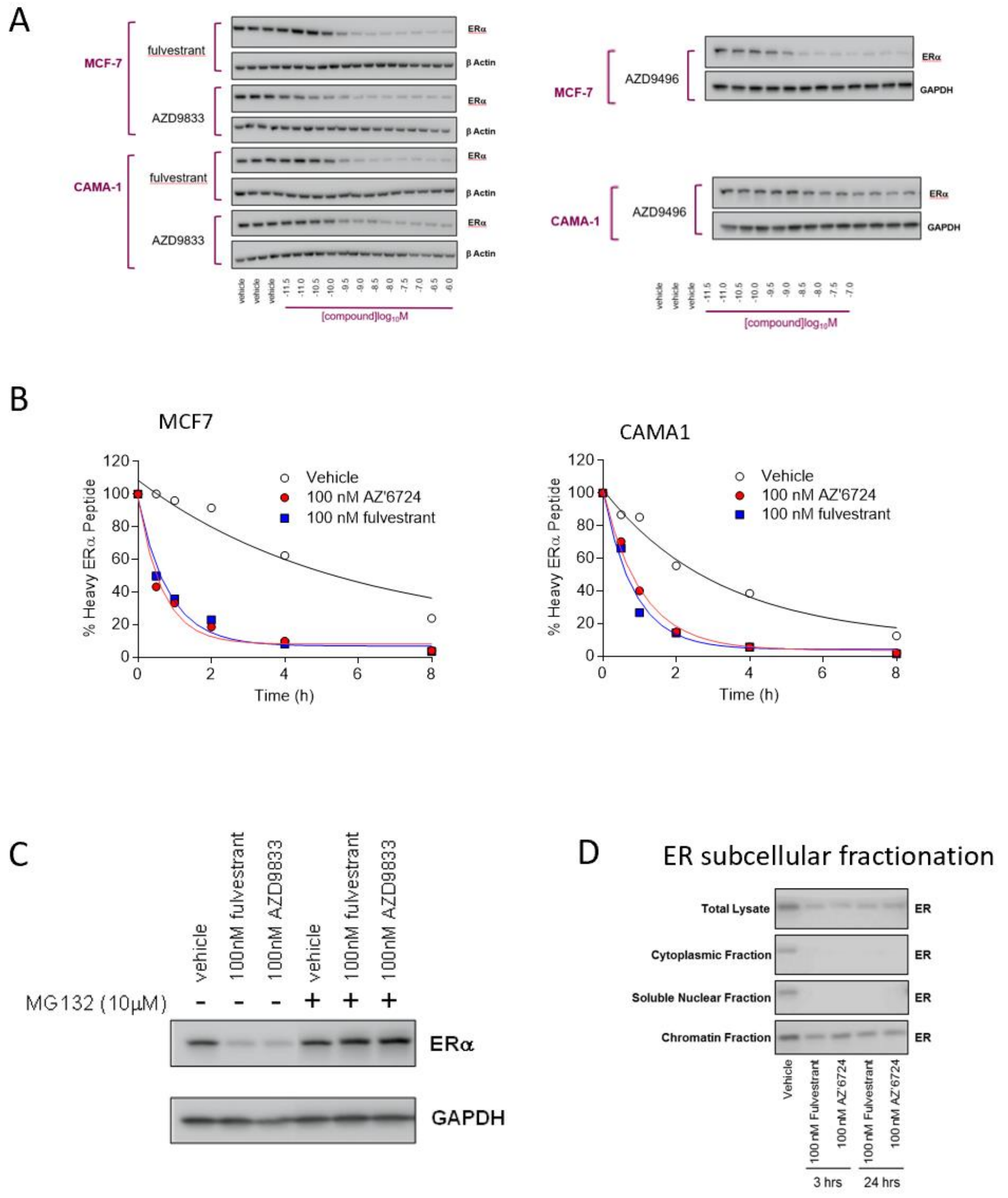

A) MCF7 or CAMA-1 cells were treated with DMSO or the indicated concentration of fulvestrant, camizestrant (AZD9833), or AZD9496 for 48 hours. ERα expression was determined by western blot. **B)** MCF7 and CAMA-1 cells cultured in the presence of heavy L-arginine were switched to media containing light L-arginine plus vehicle (0.1% DMSO) or 100 nM camizestrant (shown on graph as AZ'6724) or fulvestrant at start (T_0). At the indicated time point, cells were collected and the proportion of ERα containing heavy L-arginine was assessed by mass spectrometry. Individual data points from three independent experiments are presented with a one-phase decay line of best fit. **C)** MCF7 cells were pre-incubated for 1 hour with 10 µM of MG132. Vehicle or 100 nM camizestrant (AZD9833) or

fulvestrant was then added to the MG132-containing media and incubated with cells for a further 5 hours. The level of ERα was assessed by western blotting, with GAPDH acting as a control. **D)** MCF7 cells were incubated with vehicle (DMSO) or the indicated concentration of fulvestrant or camizestrant (shown in figure as AZ’6724) for 3 or 24 hours. Lysates were fractionated into cytoplasmic and nuclear fractions and assessed by western blot.

ER, estrogen receptor; DMSO, dimethyl sulfoxide; GAPDH, glyceraldehyde-3-phosphate dehydrogenase

Supplementary Figure 3. Binding and activity of camizestrant in clinically relevant ERα mutations

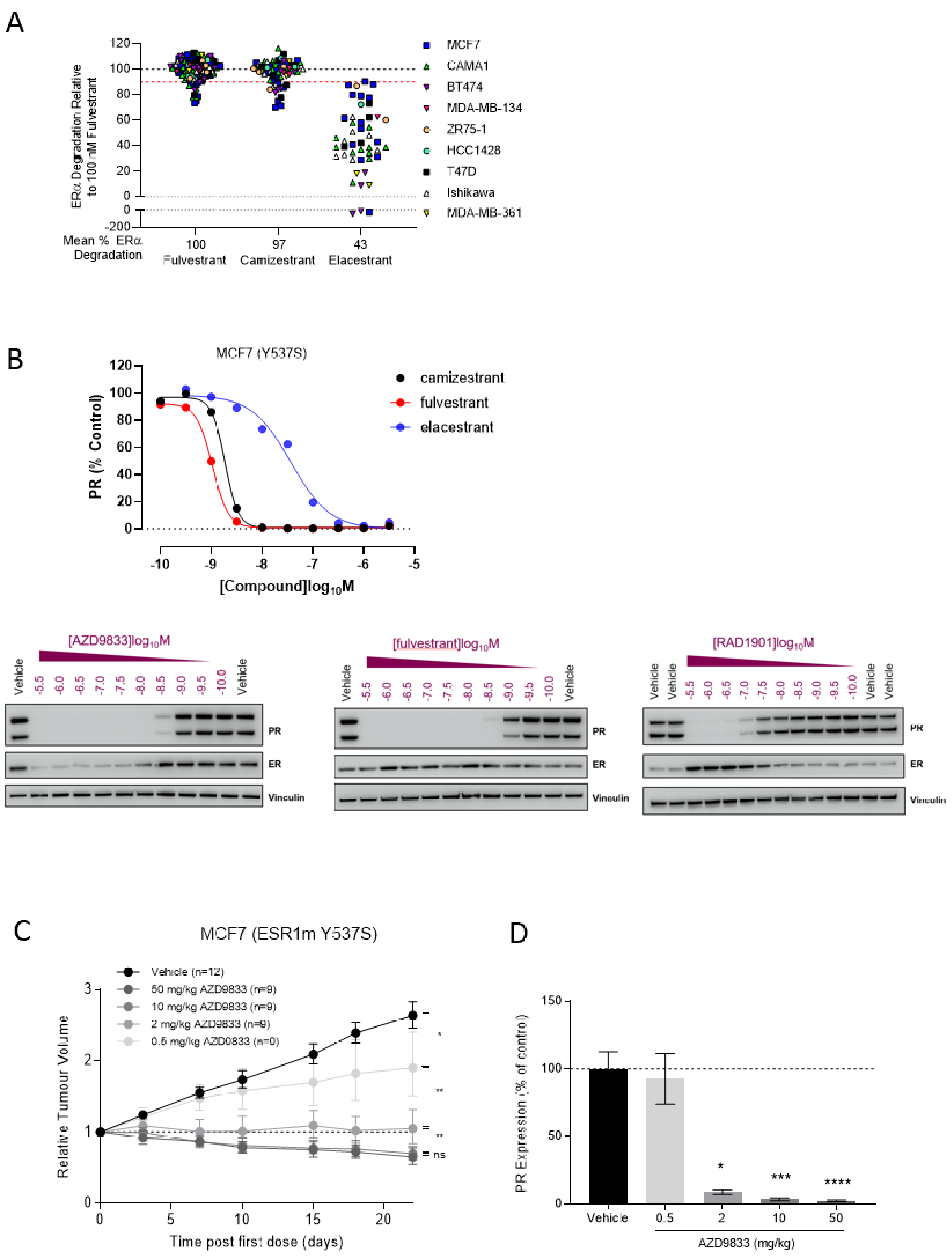

A) ERα degradation in a panel of *ESR1*wt cell lines and **B)** ERα and PR MCF7 *ESR1*m Y537S

treated at 100 nM camizestrant, fulvestrant, and elacestrant (RAD1901) for 48 hr, measured by

western blot. **C)** In MCF7 cells expressing Y537S, camizestrant (AZD9833) demonstrated anti-tumor activity in a dose-dependent manner, with maximal anti-tumor activity at 10 mg/kg. **D)** Efficacy correlated with ER degradation measured by western blot from tumors taken at the end of the efficacy dosing period. Statistical analyses were performed by one-tailed, unequal variance *t*-test *versus* log (change in tumor volume) compared with vehicle control at the final day of treatment.

ns, not significant; *p<0.05; **p<0.01; ***p<0.001; **** p<0.0001

ER, estrogen receptor; *ESR1*(m), (mutated) estrogen receptor 1 gene; PR, progesterone receptor

Supplementary Figure 4. Camizestrant causes dose-dependent cytostasis or regression in PDX models

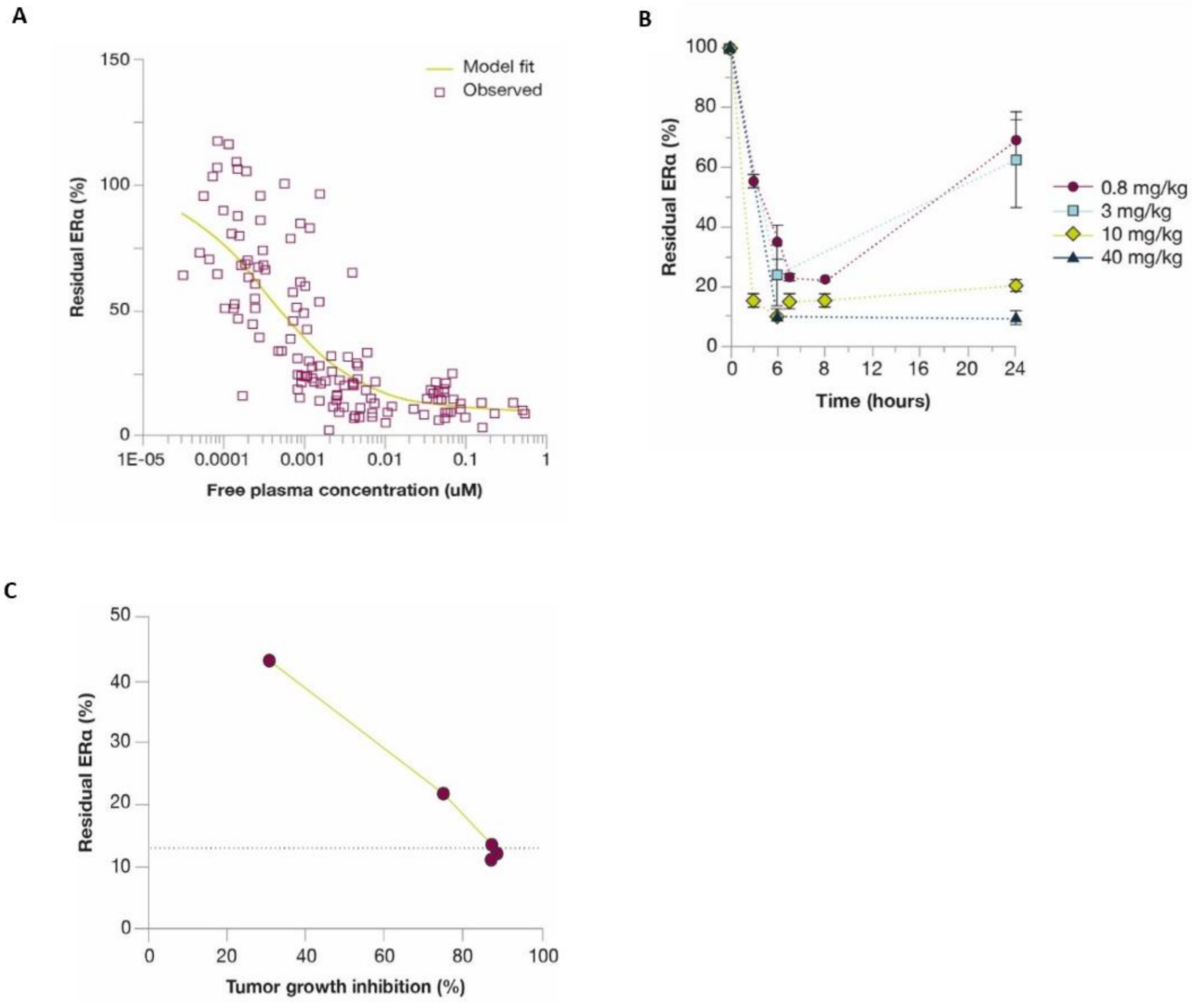

CTC174 tumors and plasma were collected at a variety of times after the last dose of camizestrant, at a variety of doses for at least 3 days. Residual ERα was determined by western blot, normalized to vehicle control, and the concentration of camizestrant in the plasma, corrected for plasma binding, was determined by liquid chromatography/mass spectrometry. **A)** Points represent observed data from individual animals, and the line describes a concentration:response relationship derived from these data. **B)** Points represent mean residual ERα (± standard deviation) from groups of tumors treated with the indicated concentration of camizestrant and collected at the indicated time after the last dose. Residual ERα was determined by western blot and immunohistochemistry and compared with vehicle control tumors. ERα immunohistochemistry was scored by H-score. Data represent individual tumors; line and error bars are geometric mean ± 95% confidence interval. **C)** Modeled mean residual ERα levels over a 28-day dosing period for camizestrant 0.8, 3, 10, 20, and 40 mg/kg

four times daily were calculated, plotted, and compared with the observed 28-day tumor growth inhibition for that dose. Points represent each camizestrant dose in ascending order. The dotted line represents 13% residual ERα, the point at which additional ERα degradation appears to have no further anti-tumor effect.

ER, estrogen receptor; PDX, patient-derived xenograft.

Supplementary Figure 5.   Camizestrant is a pure ER antagonist

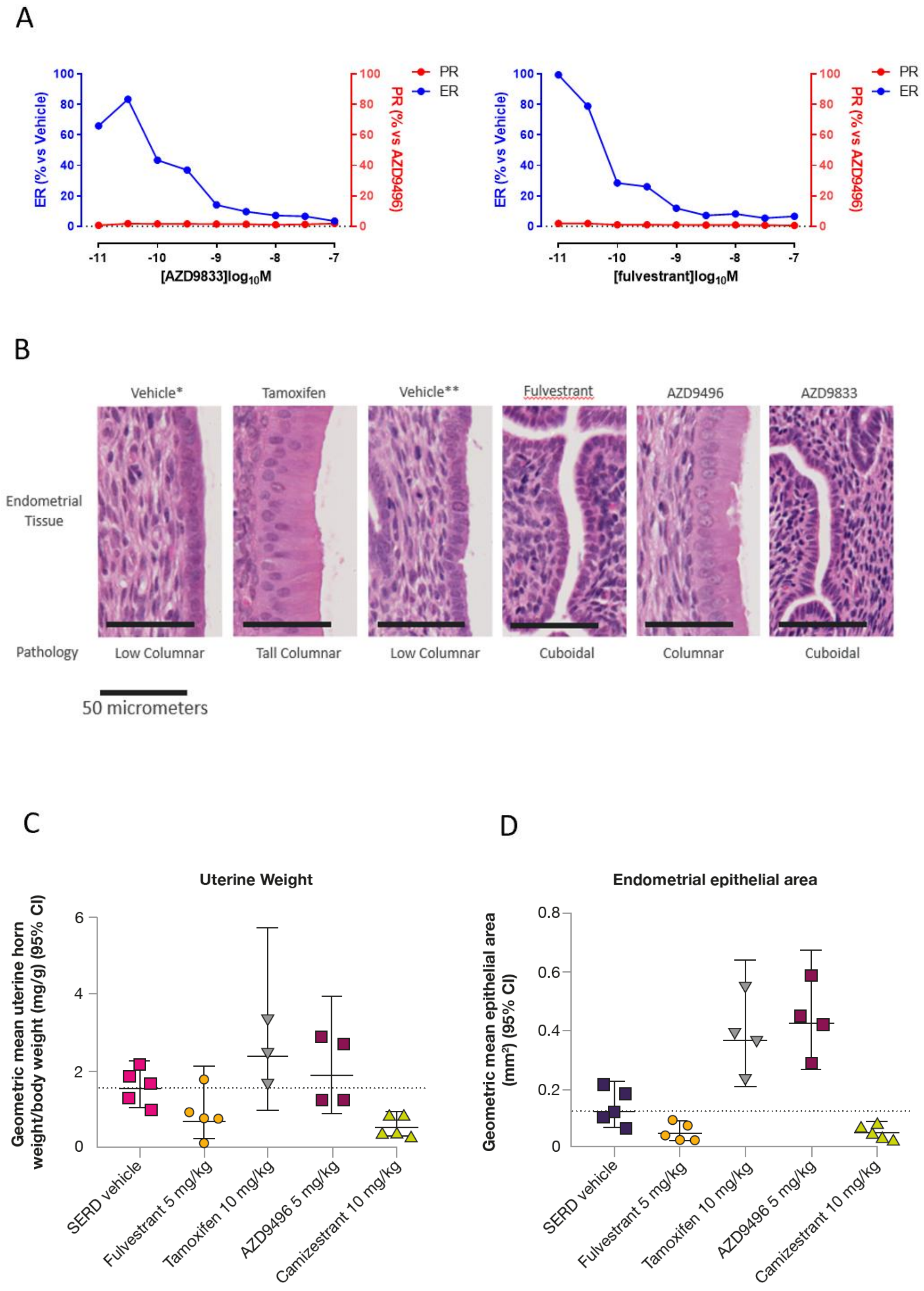

A) Ishikawa cells were incubated with camizestrant or fulvestrant for 48 hours and lysates analyzed by western blot. ER or PR protein levels are expressed relative to the AZD9496 100 nM level. **B)** Juvenile female rats were treated with the indicated dose of compound for 3 days. Twenty-four hours after the last dose, the uterus was removed. Uterine horns were processed for hematoxylin and eosin staining. The morphology of the endometrial epithelium tissue was assessed by a pathologist with representative images shown. **C, D)** The uterine horn was weighed after removal and the epithelial area was calculated via computational pathology. Data points represent individual animals. Lines represent geometric mean ± 95% CI. * Tamoxifen vehicle, ** SERDs vehicle.

CI, confidence interval; ER, estrogen receptor; PR, progesterone receptor.

Supplementary Figure 6. Camizestrant monotherapy has higher anti-tumor activity than fulvestrant in ER+ breast cancer PDX models

A

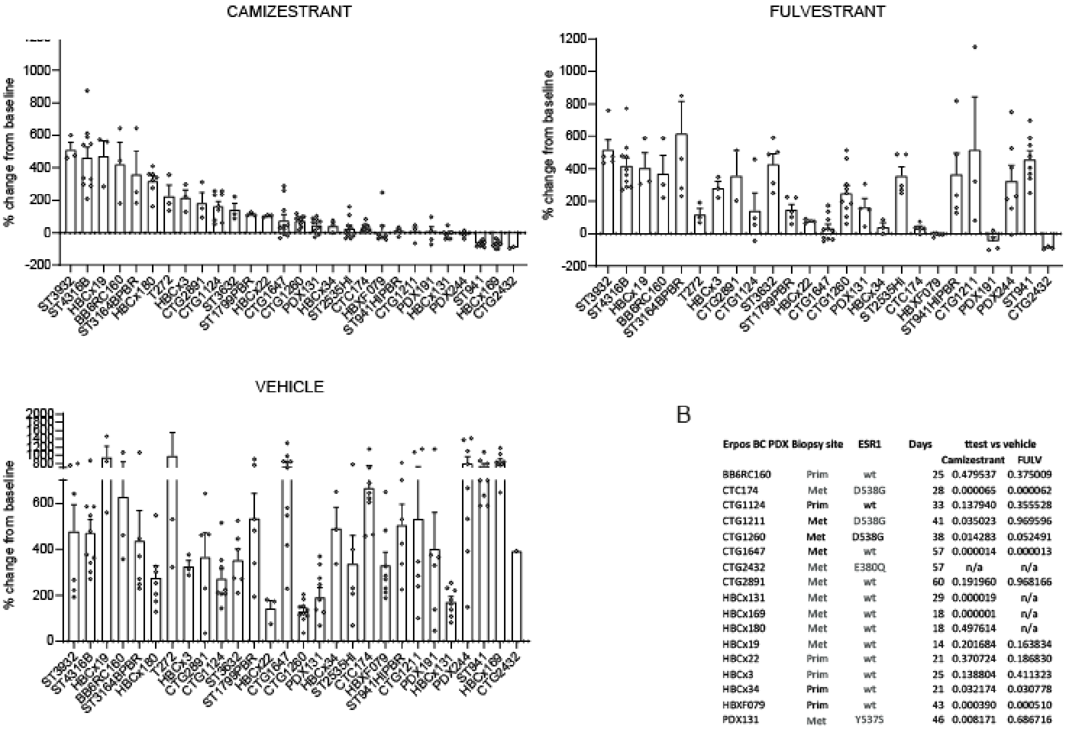

B

Erpos BC PDX	Biopsy site	ESR1	Days	ttest vs vehicle	
				Camizestrant	FULV
BB6RC160	Prim	wt	25	0.479537	0.375009
CTC174	Met	D538G	28	0.000065	0.000062
CTG1124	Prim	wt	33	0.137940	0.355528
CTG1211	Met	D538G	41	0.035023	0.969596
CTG1260	Met	D538G	38	0.014283	0.052491
CTG1647	Met	wt	57	0.000014	0.000013
CTG2432	Met	E380Q	57	n/a	n/a
CTG2891	Met	wt	60	0.191960	0.968166
HBCx131	Met	wt	29	0.000019	n/a
HBCx169	Met	wt	18	0.000001	n/a
HBCx180	Met	wt	18	0.497614	n/a
HBCx19	Met	wt	14	0.201684	0.163834
HBCx22	Prim	wt	21	0.370724	0.186830
HBCx3	Prim	wt	25	0.138804	0.411323
HBCx34	Prim	wt	21	0.032174	0.030778
HBXF079	Prim	wt	43	0.000390	0.000510
PDX131	Met	Y537S	46	0.008171	0.686716
PDX191	Prim	wt	43	0.052698	0.036801
PDX244	Met	Y537S	24	0.001477	0.027785
ST1799PBR	Met	wt	28	0.010937	0.014011
ST2535HI	Met	D538G	28	0.062550	0.879769
ST3164BPBR	Met	ESR1_CC DC170	28	0.697921	0.467423
ST3632	Prim	wt	28	0.014378	0.380675
ST3932	Prim	wt	35	0.799668	0.769040
ST4316B	Prim	wt	25	0.942185	0.645994
ST941	Met	Y537S	24	0.000002	0.002762
ST941HIPBR	Met	Y537S	28	0.002433	0.404973
T272	Prim	wt	25	0.309016	0.264348

A) Waterfall plot representing the growth of 28 PDXs treated with vehicle or camizestrant 10 mg/kg daily and 25 PDXs treated with fulvestrant 5 mg weekly. Data represents mean ± standard error of the mean, and dots represent individual values. The anti-tumor response of camizestrant *versus* fulvestrant monotherapy is represented as the percentage of tumor change compared with the initial tumor volume, benchmarked to vehicle changes. **B)** List of characteristics of the PDX models used, name, biopsy site, *ESR1* mutation status, days of treatment and p value *versus* vehicle (calculated using two-sample unequal variance *t*-test).

BC, breast cancer; ER, estrogen receptor; *ESR1*, estrogen receptor 1 gene; FULV, fulvestrant; Met, metastatic; PDX, patient-derived xenograft; Prim, primary; wt, wild-type.

Supplementary Figure 7. *Camizestrant has superior activity to fulvestrant and elacestrant activity in cell lines and* ESR1*m PDX models*

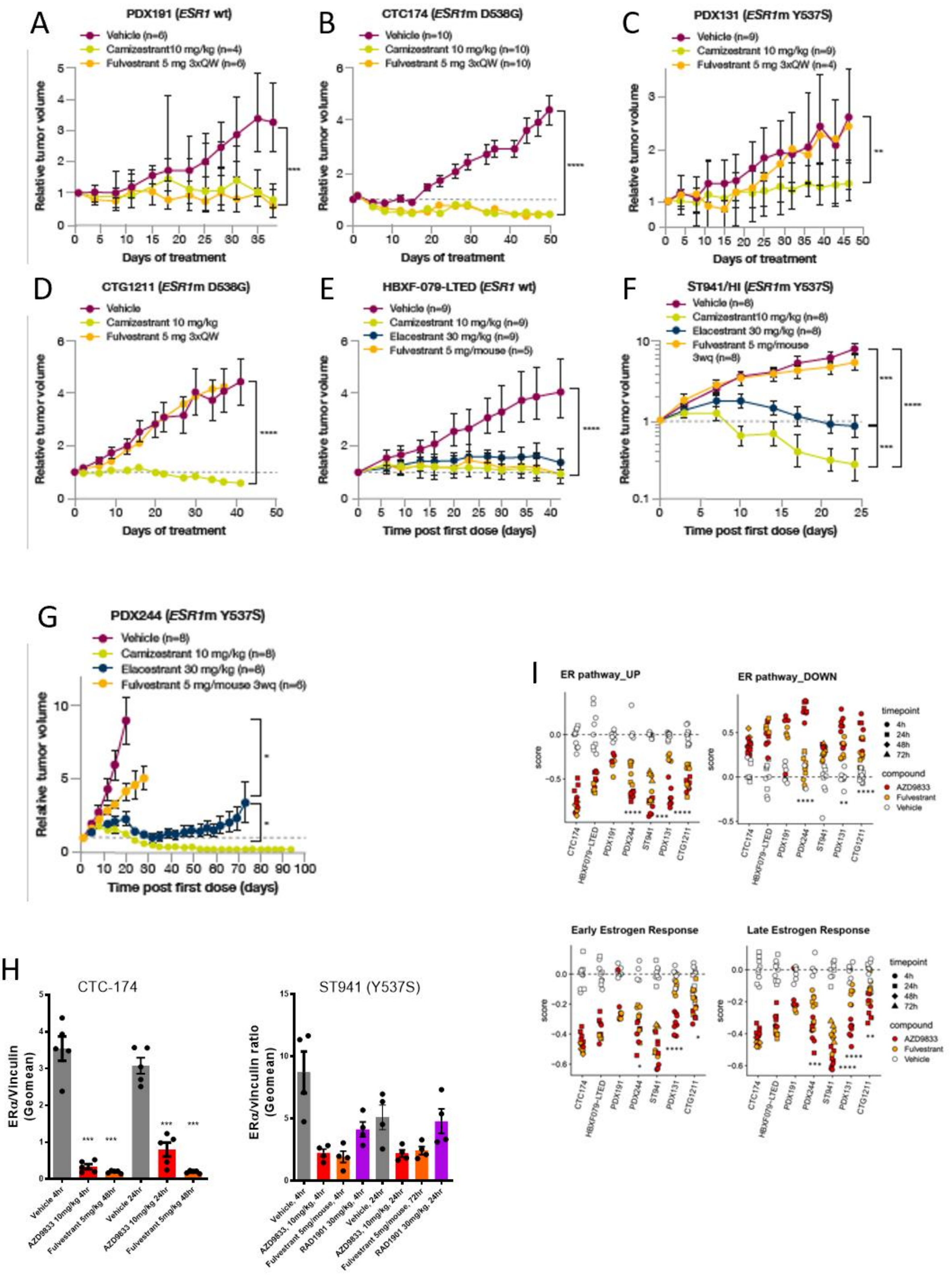

Camizestrant demonstrates enhanced efficacy compared with both fulvestrant and elacestrant in the

A) *ESR1*wt PDX191; **B)** D538G *ESR1*m PDX CTC174; **C)** Y537S *ESR1*m PDX131; **D)** D538G

*ESR1*m PDX CTG1211; **E)** *ESR1*wt PDX HBXF-079-LTED; **F)** Y537S *ESR1*m PDX ST941/H1; and **G)** Y537S *ESR1*m PDX244. Statistical analysis was performed by one-tailed, unequal variance *t*-test *versus* log (change in tumor volume) compared with vehicle control on the final day of treatment. **H)** ERα protein levels measured by western blot at indicated timepoint after drug treatment in CTC174 and ST941 PDX models. **I)** ER pathway activity in a panel of *ESR1*wt and mutant PDX models. RNA sequencing was carried out on tumors from the following PDX models treated with vehicle control or camizestrant 10 mg/kg four times daily, or fulvestrant 5 mg three times weekly, collected at the indicated time after the last dose: CTC174 (D538G), HBXF079LTED (wild-type), PDX191 (wild-type), PDX244 (Y537S), ST941 (Y537S), PDX131 (Y537S) or CTG1211 (Y537S). Data shown as change in ER pathway gene activation after treatment, expressed as change in ER pathway gene score. Plots are divided based on genes upregulated (UP) or downregulated (DOWN) by estradiol treatment. Statistical analysis comparing fulvestrant and AZD9833 was performed using one-way analysis of covariance (n≥4 animals per group).

*p<0.05; **p<0.01, ***p<0.001; ****p<0.0001

3xQW/3wq, three times weekly; ER, estrogen receptor; *ESR1*m, mutated estrogen receptor 1 gene; wt, wild-type; PDX, patient-derived xenograft

Supplementary Figure 8. Combination activity of camizestrant in ESR1wt *and CDK4/6i-insensitive ER+ breast cancer PDX models, representative of adjuvant setting*

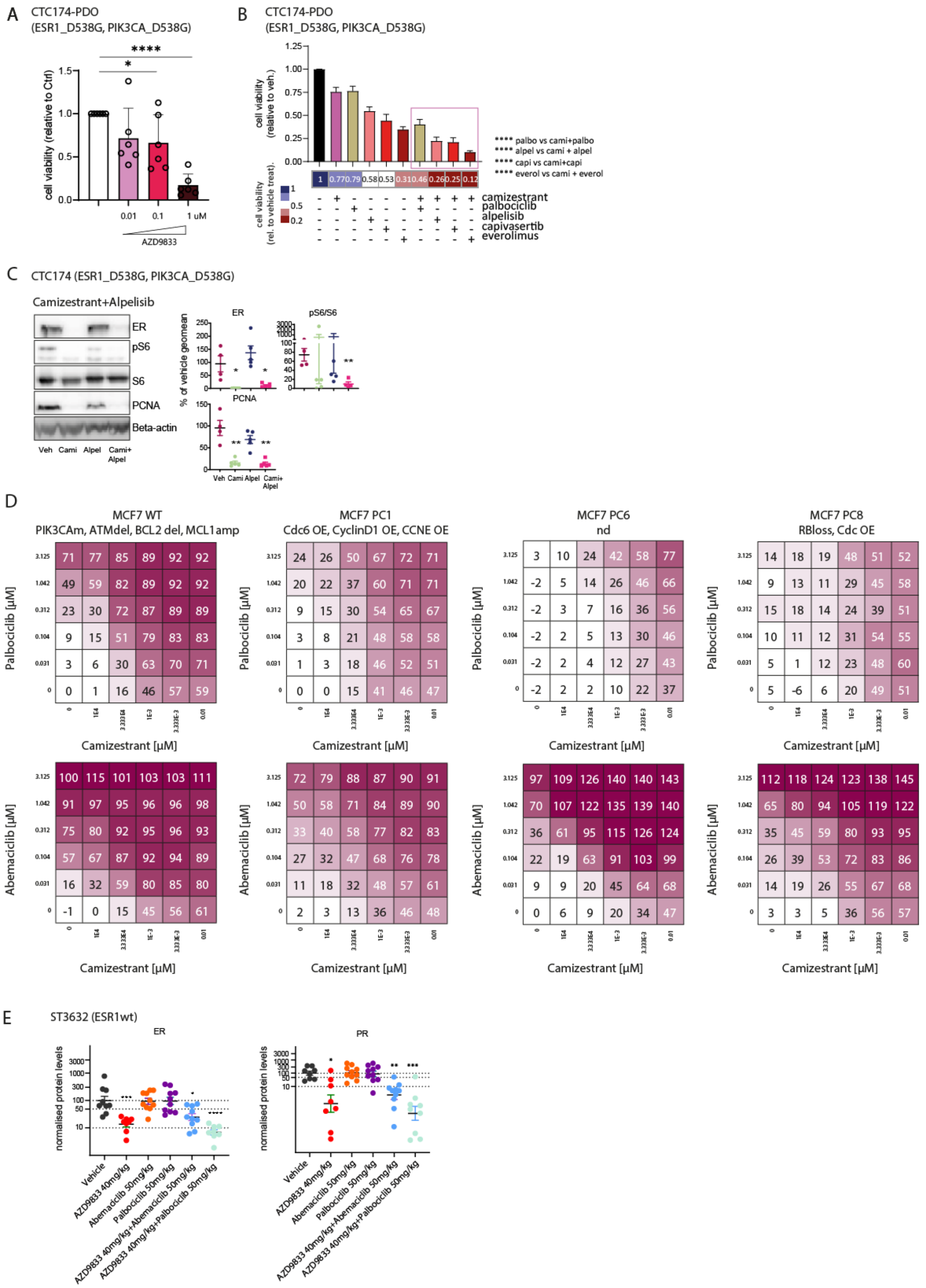

A) Activity of camizestrant in CTC174 PDX organoid models. **B)** Viability of CTC174 PDX-derived organoids treated with the indicated drugs for 144 hours. Mean ± standard error of the mean (one experiment using six independent CTC174 organoid lines in three technical replicates). P values were calculated using two-tailed paired *t* tests of biological replicates. **C)** Immunoblots of CTC174 PDX tumors and quantification of ER, pS6, S6, or PCNA following treatment. Mean ± standard deviation (n=5). p values were calculated using ANOVA tests. **D)** Genetic backgrounds for cell lines with putative mechanism of resistance to palbociclib indicated for each cell line. Combination matrix plots in MCF7 parental and palbociclib-resistant cell lines (PC1, PC6, PC8) treated with camizestrant and palbociclib or abemaciclib at indicated doses for 7 days using Cell Titer Glo viability assay. Values represent measured cell viability normalized to Day 0 (treatment day) and dimethyl sulfoxide control at Day 7. Values between 0 and 100 represent percent growth inhibition and values greater than 100 represent cell death. **E)** ST3632 PDX tumors were collected at Day 28 after treatment with camizestrant 40 mg/kg daily, palbociclib 50 mg/kg daily, and abemaciclib 50 mg/kg daily. ER and PgR protein levels measured by western blot. Statistical analysis was performed by one-tailed, unequal variance *t*-test *versus* log (change in tumor volume) on the final day of treatment ($n \geq 8$ animals per group).

*p<0.05; **p<0.01; ***p<0.001; ****p<0.0001

Alpel, alpelisib; ATM, ataxia teleangiectasia mutated; amp, amplification; BCL2, B-cell lymphoma 2; Cami, camizestrant; Capi, capivasertib; CCNE, Cyclin E; Cdc6, cell division cycle 6; CDK4/6(i), cyclin-dependent kinase 4/6 (inhibitor); Ctrl, control; del, deletion; ER, estrogen receptor; *ESR1*, estrogen receptor 1 gene; Everol, everolimus; MCL1, induced myeloid leukemia cell differentiation protein; mut, mutation; nd, not determined; Palbo, palbociclib; PCNA, proliferating cell nuclear antigen; PDX, patient-derived xenograft; PgR, progesterone receptor; PIK3CA, phosphatidylinositol 3-kinase subunit α; Rb, retinoblastoma; S6, Ribosomal protein S6; veh, vehicle; wt, wild-type

Supplementary methods

Camizestrant synthesis

Camizestrant was identified following structure-based design of a series of tricyclic indazoles, together with medicinal chemistry optimization, strategically focused on controlling lipophilicity to enhance the compound's oral bioavailability and elimination half-life.(1)

The medicinal chemistry optimization of camizestrant and full synthetic procedures have been described previously.(1) The same article also describes the experimental details of the protein–ligand crystal structure, which have been deposited in the Cambridge Crystallographic Database (https://www.rcsb.org/structure/6ZOR [accession code: 6zor]).

Stable isotope labeling of amino acids and mass spectrometry

MCF7 cells were grown for at least two passages in Rowell Park Memorial Institute (RPMI) medium modified for SILAC (R1780, Sigma) containing 10% dialyzed fetal bovine serum (FBS) and 0.1 mg/mL ^{13}C6 ^{15}N4 (heavy) L-arginine to ensure maximal labeling of proteins. Cells were cultured at 1.2×10^6 cells/well in 6-well plates (37°C; 5% CO_2) in media as before, except that 5% dialyzed charcoal-stripped FBS was used to remove endogenous estrogens. To track the degradation rate of heavy--labeled ER, media was switched to RPMI medium containing unlabeled (light) L-arginine and either camizestrant (100 nM-) or vehicle control (0.1% dimethyl sulfoxide [DMSO]). Cells were incubated for 0–48 hours (37°C, 5% CO_2), with some wells (controls) left in heavy assay media and not switched to light.

Equal protein concentrations of lysates were spiked with the same concentration of medium L-arginine (prepared lysate from MCF7 cells fully labeled with ^{13}C6 L-arginine) and immunoprecipitated overnight at 4°C on Nunc Maxisorp plates pre-coated with protein A/G and anti-estrogen receptor (ER) antibody (HC-20, Santa Cruz), followed by tryptic protein digestion.

The ER proteotypic peptide selected for relative quantification was the ligand-binding domain peptide LLFAPNLLLDR (aa402 to aa412). Analysis of this peptide was achieved by nanoflow liquid chromatography using an ultra-performance liquid chromatography M-Class System (Waters) and a

Xevo TQS triple quadrupole mass spectrometer (Waters) operating in multiple-reaction monitoring mode. The resulting chromatographic peaks were integrated in TargetLynx (Waters).

Degradation half-life was measured using the one-phase exponential decay equation in GraphPad Prism (Y = Span.e−K.X + Plateau), where X is time and Y is response, which starts out as Span + Plateau and decreases to Plateau with a rate constant K.

Viability assays

Seven-day cell viability assays for combination treatments with camizestrant and palbociclib or abemaciclib in MCF7 parental and palbociclib-resistant cell lines (PC1, PC6, PC8) were measured using Cell Titer Glo (CTG, Promega). Cells were seeded in 60 µL medium 1 day before treatment. Assay plates were dosed the next day (Day 0) and read on Day 7. An untreated plate was read on Day 0. These results were obtained with the addition of 30 µL of CTG and read for luminescence after incubation for 30 minutes at ambient temperature. Data were normalized to the original Day 0 seeding and the maximum Day 7 growth (DMSO only). Combination analysis resulting in the "matrix plots" heat maps was performed using GeneData Screener.

Juvenile rat uterine model

Female Wistar Han® rats (Envigo, Netherlands) – 16 days old at delivery, 21 days old at the start of the experiments – were used for this study. Animals were acclimatized to the laboratory conditions for at least 5 days prior to the start of experiments. Pups were housed with their dam in polysulfone type Sealsafe plus GR900 cages (Tecniplast, Lyon, France) on a bed of wood chips (Souralit, Girona, Spain) with free access to food (Rodent Maintenance Diet A04 from Safe) and water (0.2 µm filtered water). Species-appropriate environmental enrichment (Tunnel, Plexx, Uden, Netherlands) was added to the cages. The animal house was maintained under artificial lighting between 07:00–19:00 in a controlled ambient temperature of 22±2°C, and relative humidity was maintained at 55±10%. Pups were separated from their dam 1 day before the experiment start.

All test compounds were formulated 3 days before the start of the treatment period. Fulvestrant was formulated at 1 mg/mL in peanut oil; tamoxifen at 2 mg/mL in Tween 80 (1%); and camizestrant at 10 mg/mL by dissolution in 40% of the final volume of tetraethylene glycol, then addition of 50% of the final volume of Captisol® (12.5% volume/volume [v/v]), followed by pH adjustment to 3–7.5 by addition

of 250 µL of 1 M hydrochloric acid. The solution was then made up to the final volume with Captisol (12.5% v/v).

Before the experiment, animals were assigned to the different treatment groups (five animals per group, per cage) according to body weight, ensuring no significant difference between mean group body weights. Animals from the same litter were assigned to different groups.

Test compounds and respective vehicles were administered orally (PO) or subcutaneously once daily for 3 days, with a 24-hour interval (±1 hour). Fulvestrant was dosed subcutaneously at 5 mg/kg at a volume of 5 mL/kg. Tamoxifen was dosed at 10 mg/kg PO at a volume of 5 mL/kg. Camizestrant was dosed at 50 mg/kg PO at a volume of 5 mL/kg.

Animals were euthanized 24 hours after the last dose, and the uterus excised and removed from the cervix, with the horns still connected (sampling just above the cervix), and all visible excess fat was removed. A small incision was made around half-way along each horn, the horns were blotted on filter paper to remove fluids, and their weights recorded.

Individual uterus weights were entered into an Excel® spreadsheet; all data entry was cross-checked by two people. Individual uterus weights were then normalized to rat body weight by dividing uterus weight by body weight.

Tissues sections were analyzed with HALO image analysis software (Indica Labs v3.4). To determine the percentage of epithelial cells, the tissue was segmented using a DenseNet classifier into two classes: uterus and epithelium. Percentage of individual animals' epithelial area was calculated.

Cell lines and culture

All AZ cell lines were obtained from ATCC. Cells were tested and authenticated by short-tandem repeat (STR) analysis by IDEXX BioAnalytics (Columbia, USA) as follows: MCF-7 (June 2016), CAMA-1 (July 2017), T47D (December 2016), BT474 (January 2016), ZR-75-1 (January 2013), MDA-MB-361 (February 2014), Ishikawa (October 2013) and MCF-7 Y537S cells (November 2015), Mycoplasma testing was carried out by IDEXX BioAnalytics as follows: MCF-7 (June 2016), CAMA-1 (July 2017), T47D (December 2016), BT474 (January 2016), ZR-75-1 (September 2017), MDA-MB-361 (February 2014), Ishikawa (October 2013) and Y537S *ESR1*m MCF7 cells (January 2015),

The CRISPR knock-in Y537S *ESR1*m MCF7 cell line was generated as described elsewhere.(2) Cells were used within 20 passages between thawing and use in the described experiments.

Binding to ERα protein

Binding to recombinant ERα–LBD (glutathione-S-transferase [GST]; PV4543, Invitrogen) was assessed in competition assays using a LanthaScreen™ Time-Resolved Fluorescence Resonance Energy Transfer (TR-FRET) detection endpoint.

A terbium-labeled anti-GST antibody (PV3551, Invitrogen) was used to indirectly label the receptor by binding to its GST tag, and competitive binding was detected by a test compound's ability to displace a tracer fluorescent ligand (Fluormone ES2; P2645, Invitrogen) from the ERα–LBD (GST) resulting in a loss of TR-FRET signal between the terbium–anti-GST antibody and the tracer. The assay was performed with all reagents added using the Beckman Coulter BioRAPTR FRD microfluidic workstation.

Formulation of the SERDs and inhibitors

Camizestrant was formulated in triethylene glycol (TEG; 40% of final dosing volume) and 12.5% Captisol® in water (60% of final volume). The vehicle control was formulated of 40% TEG and 60% Captisol at 12.5%. Fulvestrant was formulated at 50 mg/mL in peanut oil and dosed one or three times weekly as a 0.1 mL subcutaneous injection. Elacestrant was formulated by suspending in 0.5% methylcellulose. Palbociclib was formulated by dissolving in 1% polysorbate 80. Abemaciclib was formulated in 1% hydroxyethyl cellulose, 25 mM sodium phosphate, pH 2. Everolimus was formulated by suspension in 0.5% hydroxypropyl methylcellulose/0.1% Tween 80. Capivasertib was formulated by dissolving in DMSO (10% of final dosing volume), 1 M hydrochloric acid (2% of final dosing volume) and 25% kleptose (80% of final volume). The solution was adjusted to pH 5.0 (±0.1) then made up to final volume with 25% kleptose. Alpelisib was formulated in 0.5% hydroxypropyl methylcellulose/0.1% Tween 80. All oral (PO) dosing solutions were replaced every 7 days and stored at room temperature in bottles protected from light.

Patient-derived tumor models (PDX)

For the Y537S ESR1m MCF7 xenograft, 5x106 cells in 100 µL were implanted subcutaneously with Matrigel 1:1 in severe combined immunodeficiency (SCID) male mice (Envigo UK). Tumor volume

(TV) was calculated using the formula: TV (mm3) = (3.142 x max (length:width) x min (length:width))/6,000.

For the camizestrant dose–response study in the CTC174 PDX model (hormone-independent), established tumors were removed from two donor mice and dissected into 3x3x3 mm pieces in RPMI 1640 media. Female NOD SCID gamma (NSG) mice (surgically ovariectomized at Jackson Laboratories, Bar Harbor, ME, USA) were anesthetized using 2% isoflurane. A small skin incision was made 0.5 cm dorsally to the third nipple, and a tumor fragment was inserted between the skin layer and the third mammary fat pad. The skin was closed with surgical glue (VetBond, 3M). TV was calculated as TV (mm3) = width2 x length x 0.52.

For the combination studies in the CTC174 PDX model (hormone-independent), established tumors were removed from donor mice and dissected into 3x3x3 mm pieces in RPMI 1640 media. Female NSG mice (CRL UK) were anesthetized using 2% isoflurane. A small skin incision was made 0.5 cm dorsally to the third nipple, and a tumor fragment was inserted between the skin layer and the third mammary fat pad. The skin was closed with surgical glue (VetBond, 3M). TV was calculated using the formula: TV (mm3) = (3.142 x max (length:width) x min (length:width) x min (length:width))/6,000, or (mm3) = width2 x length x 0.52 for the CTC174 study.

For studies in the HBXF079-LTED PDX model (hormone-independent), established tumors were removed from donor mice and re-implanted between the skin layer and the third mammary fat pad in immunocompromised female NSG mice. Tumor volume was calculated as TV (mm3) = width2 x length x 0.52. The vehicle control or camizestrant at 10 mg/kg were dosed PO four times daily for 42 days at 0.1 mL per 10 g of mouse (n=8/9 per group). Tumors were harvested and snap frozen in liquid nitrogen 24 hours after the last dose.

For studies in the ST941 and ST3164B (hormone-independent), and ST1799, ST3932, and ST3632 (exogenous hormone-requiring; XenoSTART) PDX models, established tumors were removed from donor mice and re-implanted unilaterally on the left flank in immunocompromised ovariectomized athymic nude (Crl:NU(NCr)-Foxn1nu) mice (XenoSTART). TV was calculated as TV (mm3) = width^2 x length x 0.52.

For the CTG-1211 and CTG-2432 models (Champions Oncology) stock mice were produced in athymic nudes (plus estrogen supplementation). Established tumors were removed from donor mice and re-implanted unilaterally on the left flank in immunocompromised athymic nude mice. TV was calculated as TV (mm3) = width^2 x length x 0.52.

For the PDX131, PDX191, and PDX244 models (VHIO), NMRI mice (6-week-old female athymic HsdCpb:NMRI-Foxn1nu; Harlan Laboratories) were implanted for each assay (efficacy and pharmacodynamic [PD] analysis). TV was calculated using the ellipsoid formula: TV = (length x width2) x (π/6). All implants came from the same tumor for each PDX. PDX191 and PDX244 animals were continuously supplemented with 1 µM 17β-estradiol (Sigma-Aldrich) in their drinking water.

For the HBCx131, HBCx180 and HBCx169 models (Institute Curie), 140 mice (8 week-old female Swiss nude mice, Charles River Laboratories) were implanted for each efficacy assay. All implants came from the same tumor for each PDX. Animals grafted with HBCx131 and HBCx180 were continuously supplemented with 17β estradiol (Sigma-Aldrich) in their drinking water (2 µg/ml until start of treatment, 0.5 µg/ml after start of treatment). TV was calculated using the formula: TV = a x b2/2, where a is the largest diameter and b is the smallest diameter.

For the BB6RC160, HBCx3, HBCx19, HBCx22, HBCx34, and T272 models (XenTech), 5–11-week-old female athymic HsdCpb:NMRI-Foxn1nu mice (Envigo) were implanted for each efficacy assay (three mice per group). TV was calculated using the formula: TV (mm3) = [length x width2]/2. All implants came from the 3 to 24 donor tumors for each PDX, with no supplementation, except for the BB6RC160 model that received estrogen diluted in drinking water (β-oestradiol, 8.5 mg/l), from the date of tumor implant to the end of the study.

Patient-derived tumor cells (PDTCs) – CTC174 organoids

Tissues from CTC174 xenografts were treated with 0.1% collagenase I (ThermoFisher Scientific 17018029) for 4–6 hours in Dulbecco′s Modified Eagle′s Medium (DMEM)/F12 (1:1 ratio, ThermoFisher Scientific 31330038) containing penicillin–streptomycin (1% ThermoFisher Scientific) and normocin (InvivoGen ant-nr-05) at 37°C with gentle agitation. The reaction was stopped by adding 10% heat-inactivated FBS (ThermoFisher Scientific A3840001), and the solution was filtered using a 70 µm cell strainer. Cells were collected by centrifugation (500 rpm for 5 minutes) and grown

in suspension in DMEM/F12 (1:1 ratio, ThermoFisher Scientific 31330038) culture media supplemented with B27 (ThermoFisher Scientific, 17504044), basic epidermal growth factor (10 ng/mL, ThermoFisher Scientific PHG0266), fibroblast growth factor (10 ng/mL, ThermoFisher Scientific PHG0315), heparin (4 µg/mL, Sigma H3149-10KU) with penicillin–streptomycin (1%) and normocin (1%).

The resulting organoids were digested to perform cell viability assays. Organoids were pelleted at 500 rpm for 5 minutes in 15 mL tubes. After aspirating the supernatant, pellets were treated with TryPLE (ThermoFisher Scientific A1217701) for 5 minutes, 3 mL culture media was added, and tubes were centrifuged at 500 rpm for 5 minutes. Single cells were then seeded onto 96-well plates at 0.04–0.1x10^6 cells/mL in culture media in suspension, and analyzed after drug treatment for 144 hours. Cells were treated with 10% alamarBlue (ThermoFisher DAL1025) and then incubated for 6 hours at 37°C. Two to three biological replicates of experiments were performed for six lines disassociated from six different CTC174 xenografted mice in three to four technical replicates. Fluorescence was excited at 570 nm (PerkinElmer Multimode Plate Reader Envision). Drug concentrations were refreshed every 48 hours: camizestrant (0.1 µM), fulvestrant (0.1 µM), palbociclib (1 µM), alpelisib (1 µM), capivasertib (AZD5363; 1 µM), and everolimus (0.1 µM).

In vitro gene expression analysis in cell lines and PDX models

For Figure 4B (ER-pathway activity), data are shown as change in ER pathway gene activation after treatment, expressed as change in ER pathway gene score, as described previously.(3) In summary, the fold-change of estradiol genes in relation to the baseline condition (vehicle) was calculated per sample. Then fold-changes were averaged separately across estradiol-induced and repressed genes. Finally, the ER pathway score was obtained by subtracting the estradiol-repressed from the estradiol-induced score. All other pathway scores (for Figure 4B [Cell cycle G1-S pathway] and Supplementary Figure 6I) were calculated using GSVA, as previously described.(4) For visualisation purposes, scores for each model were then centered around the median of the respective vehicle. GSVA scores were calculated and used to assess changes in expression of genes differentially expressed in early response to estrogen (Hallmark_ESTROGEN_EARLY GSEA/MSigDB); differentially expressed in late response to estrogen (Hallmark_ESTROGEN_LATE GSEA); and regulating cell cycle G1/S checkpoint (genes: *ABL1, ATM, ATR, CCNA1, CCND1, CCNE1 CDC25A, CDK1, CDK2, CDK4,*

CDK6, CDKN1A, CDKN2A, CDKN2B, DHFR, E2F1, GSK3B, HDAC1, RB1, SKP2, SMAD3, SMAD4, TFDP1, TGFB1, TGFB2).

Western blot

To determine ER and PgR protein levels in end-of-study tumor samples, tumor fragments were added to 700 µL of Invitrogen cell extraction buffer (FNN0011) with added Sigma phosphatase inhibitors (No.2 [P5726] and No.3 [P0044]; 1:100 dilution), protease inhibitor cocktail (P8340; 1:200 dilution), and Roche complete protease inhibitor (11836145001; one tablet per 50 mL). Samples were homogenized for 20 seconds using a T25 Ultra-Turrax homogenizer, then centrifuged at 13,000 rpm for 15 minutes. Protein levels in the supernatant were then measured, and approximately 45 µg of protein was run on a 4–12% bis-tris gel using standard methods. For other markers, the protein extracted by adding 900 µL of Extraction buffer (20mM Tris (ph 7.5) (Sigma T2319), 137mM NaCl (Sigma S5150), 10% Glycerol (Sigma G5516), 50mM NaF (Sigma S6776), 1mM Na_3VO_4 (Sigma S6508), 1% SDS, 1% NP40 subsitute (Roche 11754599001) with complete protease inhibitor cocktail (Roche 11836145001; 1 tablet per 50 mL) and phosphatase inhibitor cocktail #3 (Sigma P0044) with benzoase nuclease (Sigma E1014). Samples were homogenized for 30 seconds three times at 6.5m/s in fast-prep machine with an incubation at 4oC for 5min between runs. Lysates were then sonicated in chilled diagenode bioruptor in chilled water bath for five cycles of 30sec on high / 30sec off. Lysates were then centrifuged for 10min at 13000rpm at 4oC for two times, with a change of tube between runs to discard debris. Lysates were transferred into a new tube, and protein in the supernatant measured (Thermofisher 23227).

Approximately 40µg of protein was run on a 4–12% bis-tris gel using standard methods. Following protein separation, protein was transfer onto nitrocellulose membranes (Iblot2 Thermofisher #IB21001). Primary antibodies were diluted in tris-buffered saline (TBS)/0.05% Tween (TBST) + 5% Marvel, and incubated overnight at 4°C; anti-ERα antibody (ThermoFisher SP1 #9101S; 1:400 dilution), anti-PgR (Dako PgR636; 1:200 dilution), pRb1 S807/811 (CST #8516 1:1000 dilution), Rb1 (CST #9309 1:1000 dilution), pAKT S473 (CST #4060 1:1000 dilution), AKT (CST #9272 1:1000 dilution), pS6 S235/236 (CST #2217 1:1000 dilution), S6 (CST #4858 1:1000 dilution), PCNA (CST #13110 1:1000 dilution), vinculin (Sigma V931; 1:10,000 dilution), βactin (CST #4970 1:2000 dilution). The membranes were washed three times for 15 minutes each in 20 mL of TBST. A secondary rabbit

(CST #7074) or mouse (CST #7076) horseradish peroxidase (HRP)-linked antibody was diluted 1:2000 or 1:1,000 (for PgR) in TBST + 5% Marvel and incubated for 1 hour at room temperature. The membranes were washed three times for 15 minutes each in 20 mL of TBST, and signal was detected using chemiluminescent SuperSignal West Dura extended duration substrate and quantified using Syngene software. Statistical analysis was performed on values normalized to vinculin or geomean of vinculin and βactin using a one-tailed, unequal variance *t*-test compared with vehicle control or ordinary one-way ANOVA multiple compared with vehicle control, described in the figure legends. Individual animal percentage inhibitions were calculated using the following equation:

$$Individual\ animal\ percentage\ inhibition\ =\ ([]/vehicle\ control\ geometric\ mean))\ \times\ 100\%$$

References to supplement

1. Scott JS, Moss TA, Balazs A, Barlaam B, Breed J, Carbajo RJ*, et al.* Discovery of AZD9833, a Potent and Orally Bioavailable Selective Estrogen Receptor Degrader and Antagonist. J Med Chem **2020**;63:14530–59
2. Toy W, Weir H, Razavi P, Lawson M, Goeppert AU, Mazzola AM*, et al.* Activating ESR1 Mutations Differentially Affect the Efficacy of ER Antagonists. Cancer Discov **2017**;7:277-87
3. Guan J, Zhou W, Hafner M, Blake RA, Chalouni C, Chen IP*, et al.* Therapeutic ligands antagonize estrogen receptor function by impairing its mobility. Cell **2019**;178:949–63.e18
4. Hänzelmann S, Castelo R, Guinney J. GSVA: gene set variation analysis for microarray and RNA-Seq data. BMC Bioinformatics **2013**;14:7
